# The photoreactivation of 6 − 4 photoproducts in chloroplast and nuclear DNA depends on the amount of the Arabidopsis UV repair defective 3 protein

**DOI:** 10.1186/s12870-024-05439-0

**Published:** 2024-07-30

**Authors:** Piotr Zgłobicki, Paweł Hermanowicz, Kinga Kłodawska, Aneta Bażant, Justyna Łabuz, Joanna Grzyb, Małgorzata Dutka, Ewa Kowalska, Joanna Jawor, Katarzyna Leja, Agnieszka Katarzyna Banaś

**Affiliations:** 1 Department of Plant Biotechnology, Faculty of Biochemistry, Biophysics and Biotechnology, Jagiellonian University, Gronostajowa 7, Kraków, 30-387 Poland; 2 Malopolska Centre of Biotechnology, Jagiellonian University, Gronostajowa 7A, Kraków, 30-387 Poland; 3 Department of Plant Physiology and Biochemistry, Faculty of Biochemistry, Biophysics and Biotechnology, Jagiellonian University, Gronostajowa 7, Kraków, 30-387 Poland; 4 Department of Biophysics, Faculty of Biotechnology, University of Wrocław, F. Joliot-Curie 14a, Wrocław, 50-383 Poland; 5 Department of Molecular Biophysics, Faculty of Biochemistry, Biophysics and Biotechnology, Jagiellonian University, Gronostajowa 7, Kraków, 30-387 Poland; 6 Doctoral School of Exact and Natural Sciences, Jagiellonian University, prof. S. Łojasiewicza 11, Kraków, 30-348 Poland

**Keywords:** Arabidopsis, AtUVR3, Chloroplast nucleoid, Photolyase, Photoreactivation, (6 − 4) pyrimidine–pyrimidone photoproduct

## Abstract

**Background:**

6 − 4 photoproducts are the second most common UV-induced DNA lesions after cyclobutane pyrimidine dimers. In plants, they are mainly repaired by photolyases in a process called photoreactivation. While pyrimidine dimers can be deleterious, leading to mutagenesis or even cell death, 6 − 4 photoproducts can activate specific signaling pathways. Therefore, their removal is particularly important, especially for plants exposed to high UV intensities due to their sessile nature. Although photoreactivation in nuclear DNA is well-known, its role in plant organelles remains unclear. In this paper we analyzed the activity and localization of GFP-tagged AtUVR3, the 6 − 4 photoproduct specific photolyase.

**Results:**

Using transgenic Arabidopsis with different expression levels of AtUVR3, we confirmed a positive trend between these levels and the rate of 6 − 4 photoproduct removal under blue light. Measurements of 6 − 4 photoproduct levels in chloroplast and nuclear DNA of wild type, photolyase mutants, and transgenic plants overexpressing AtUVR3 showed that the photoreactivation is the main repair pathway responsible for the removal of these lesions in both organelles. The GFP-tagged AtUVR3 was predominantly located in nuclei with a small fraction present in chloroplasts and mitochondria of transgenic *Arabidopsis thaliana* and *Nicotiana tabacum* lines. In chloroplasts, this photolyase co-localized with the nucleoid marked by plastid envelope DNA binding protein.

**Conclusions:**

Photolyases are mainly localized in plant nuclei, with only a small fraction present in chloroplasts and mitochondria. Despite this unbalanced distribution, photoreactivation is the primary mechanism responsible for the removal of 6 − 4 photoproducts from nuclear and chloroplast DNA in adult leaves. The amount of the AtUVR3 photolyase is the limiting factor influencing the photoreactivation rate of 6 − 4 photoproducts. The efficient photoreactivation of 6 − 4 photoproducts in 35S: AtUVR3-GFP Arabidopsis and *Nicotiana tabacum* is a promising starting point to evaluate whether transgenic crops overproducing this photolyase are more tolerant to high UV irradiation and how they respond to other abiotic and biotic stresses under field conditions.

**Supplementary Information:**

The online version contains supplementary material available at 10.1186/s12870-024-05439-0.

## Background

Most plants are autotrophic organisms, and as such depend on solar light as an energy source for photosynthesis. Photosynthetically active radiation is inevitably accompanied by shorter wavelengths, the ultraviolet (UV), which is traditionally subdivided into UV-A (315–400 nm), UV-B (280–315 nm), and UV-C (100–280 nm) ranges. The shortest wavelengths are absorbed by the stratospheric ozone, so the UV radiation that reaches the Earth’s surface mostly comprises UV-A with a UV-B tail and is completely lacking UV-C. The remaining UV portion of the solar spectrum interacts with living organisms, being absorbed by numerous biomolecules, such as proteins, fatty acids, and nucleic acids. In DNA, direct UV absorption can lead to the formation of pyrimidine dimers, resulting from a covalent bond formation between adjacent pyrimidines. These are mainly cyclobutane pyrimidine dimers (CPDs) and pyrimidine 6 − 4 pyrimidones (6 − 4 photoproducts, 6 − 4 PPs), with a small amount of Dewar isomers created by isomerization of 6 − 4 PPs under UV-A [1–3]. Such lesions stop the progress of DNA and RNA polymerases on the DNA strand [4] and may either cause cell death if unrepaired or become mutagenic if repaired incorrectly or bypassed due to translesion synthesis [5, 6]. Pyrimidine dimers may be repaired by excision repair (reviewed in: [7]) or photoreactivation (reviewed in: [8]); however, some organisms, such as placental mammals have lost the latter repair mechanism. Photoreactivation is catalyzed by photolyases, which are flavoproteins that use UV-A or blue light to separate the bases forming the dimer [9]. Most known photolyases specifically bind only one type of dimer, but recently a protein capable of binding both CPDs and 6 − 4 PPs has been described in Antarctic *Sphingomonas* sp. [10].

In non-proliferating plant cells, photoreactivation is the most important pathway for pyrimidine dimer repair [11, 12]. CPD photolyase has been discovered in numerous plants, including Arabidopsis [13, 14], mustard [15], cucumber [16, 17], rice [18], and sorghum [16]. Although plant 6 − 4 PP photolyases are less studied, proteins with such activity have been described among others in Arabidopsis [14, 19] and the Antarctic microalga *Chlamydomonas* sp. UWO241 [20].

The major role of photoreactivation in the removal of pyrimidine dimers in plants was demonstrated by Dany and co-workers [2]. 6 − 4 PPs are less abundant than CPDs because they require higher energy to form. The CPD to 6 − 4 PP ratio reached 9 in Arabidopsis suspension culture and 2 in etiolated hypocotyls irradiated with UV-B [2, 21]. When UV exposure was followed by darkness, the growth of seedling roots was inhibited to a similar extent in Arabidopsis mutants with impaired photoreactivation of either CPDs or 6 − 4 PPs [14]. However, when UV-irradiated seedlings were cultured under photoreactivation light, UV dose required to reduce root growth by 63% was seven-fold lower in plants lacking CPD photoreactivation and only three times lower in plants defective in 6 − 4 PP photoreactivation. Similarly, Arabidopsis plants grown under conditions simulating natural UV irradiation showed a higher rate of mutations in plants devoid of CPD but not 6 − 4 PP photolyase activity [22]. It has also been shown that the photoreactivation of CPDs is one of the factors involved in the UV-resistance of rice cultivars [23]. These results indicate that the lack of effective dimer repair leads to high UV-sensitivity, with the more prominent role being the removal of CPDs. On the other hand, specific signaling pathways are triggered by 6 − 4 PPs but not by CPDs, as shown in human cells [24, 25]. This suggests that the physiological role of the 6 − 4 PPs extends beyond simply causing genotoxic effects. Although it has never been tested, it is highly possible that 6 − 4 PPs act as signaling molecules in plants as well. In such a case, the activity of 6 − 4 PP specific photolyases would be one of the components controlling the UV-induced signal strength and duration.

In addition to nuclei, eukaryotic organisms contain DNA in organelles - mitochondria and chloroplasts. Research on the repair of organellar DNA by photoreactivation is limited. This process is responsible for the repair of pyrimidine dimers in mitochondrial (mt) DNA in yeast *Saccharomyces cerevisiae* [26, 27], and in chloroplast (cp) DNA in *Chlamydomonas* [28]. Light-dependent removal of unspecified UV-induced DNA lesions has also been described in cpDNA of soybean [29] and 14-day-old Arabidopsis leaves [30]. The levels of CPDs in cpDNA and mtDNA of maize leaves were lower when illuminated with white light compared to leaves left in darkness [31]. Most current studies on plant CPD photolyases focus on rice. Using rice strains with different levels of CPD photolyase (OsPHR), Takahashi and co-workers demonstrated that this enzyme acts in CPD repair in all three DNA-containing organelles [32]. Only recently was the repair of CPDs in Arabidopsis mtDNA confirmed [33]. Contrary to these results, photoreactivation was not observed in isolated chloroplasts from spinach leaves [34] or in chloroplasts or mitochondria of 5-day-old Arabidopsis seedlings [35]. This suggests that photoreactivation in plant organelles may be species or age-specific. It is worth noting that direct data showing the photoreactivation of 6 − 4 PPs in plant organelles are not yet available. One of the aims of the current study was to fill this gap.

To date, three Arabidopsis proteins with confirmed photolyase activity have been identified. Two of them, PHOTOLYASE 1/UV RESISTANCE 2 (AtPHR1/UVR2) and CRYPTOCHROME3 (AtCRY3), repair CPDs [13, 36, 37]. 6 − 4 PPs are repaired by UV REPAIR DEFECTIVE 3 (AtUVR3) protein [14, 19]. Most of the data on plant 6 − 4 PP specific photolyases come from in vitro experiments using isolated proteins. In this study, we used plants stably expressing AtUVR3-GFP. A set of Arabidopsis lines with different amounts of AtUVR3 was tested to link the expression levels of this photolyase with the efficiency of 6 − 4 PP photoreactivation. The levels of 6 − 4 PPs in cpDNA isolated from *uvr3* mutant and overexpressor plants were compared. Interestingly, our results show that these DNA lesions were removed by AtUVR3 photolyase not only in nDNA but also in chloroplast nucleoids. Moreover, the amount of this enzyme appeared to be the limiting factor in the repair process in both organelles. To our knowledge, this is the first evidence showing directly that 6 − 4 PPs localized in chloroplast nucleoids are repaired by photoreactivation.

Subsequently, the subcellular localization of AtUVR3-GFP fusion protein in the transgenic Arabidopsis and *Nicotiana tabacum* plants was evaluated. Whereas this protein was observed in all DNA containing organelles in transiently transformed *Nicotiana benthamiana* [38], most GFP fluorescence was present in nuclei with only a weak signal visible in chloroplasts and mitochondria of our transgenic plants. However, the green signals in mitochondria and chloroplasts were stronger than autofluorescence levels observed in wild-type (WT) plants.

Finally, we examined the (sub)chloroplastic localization of AtUVR3. The observed co-localization of this photolyase with the plastid envelope DNA binding protein (PEND) [39] suggests that it was associated with the plastid nucleoid even in the absence of 6 − 4 PPs. These results open the possibility of studying the physiological role of photolyase binding to undamaged DNA and the mechanisms involved in searching for pyrimidine dimers.

## Results

In this study, we used WT and transgenic *N. tabacum* cv Samsun and *Arabidopsis thaliana* plants. In addition to these lines we also used *uvr3* and *uvr3phr1* Arabidopsis mutants. The Arabidopsis lines overexpressing AtUVR3-GFP under the 35S promoter in the WT and *uvr3* backgrounds were designated as AtUVR3GFP-1/2 and *uvr3*:AtUVR3GFP-2/6/7/11/12, respectively. Transgenic *N. tabacum* plants were marked as AtUVR3-GFP. A detailed description of all the plant lines is given in the *Plant material and culture conditions* subsection of *Materials and Methods*.

### Levels of AtUVR3 transcripts are higher when expressed under 35 S promoter

**Fig. 1:**
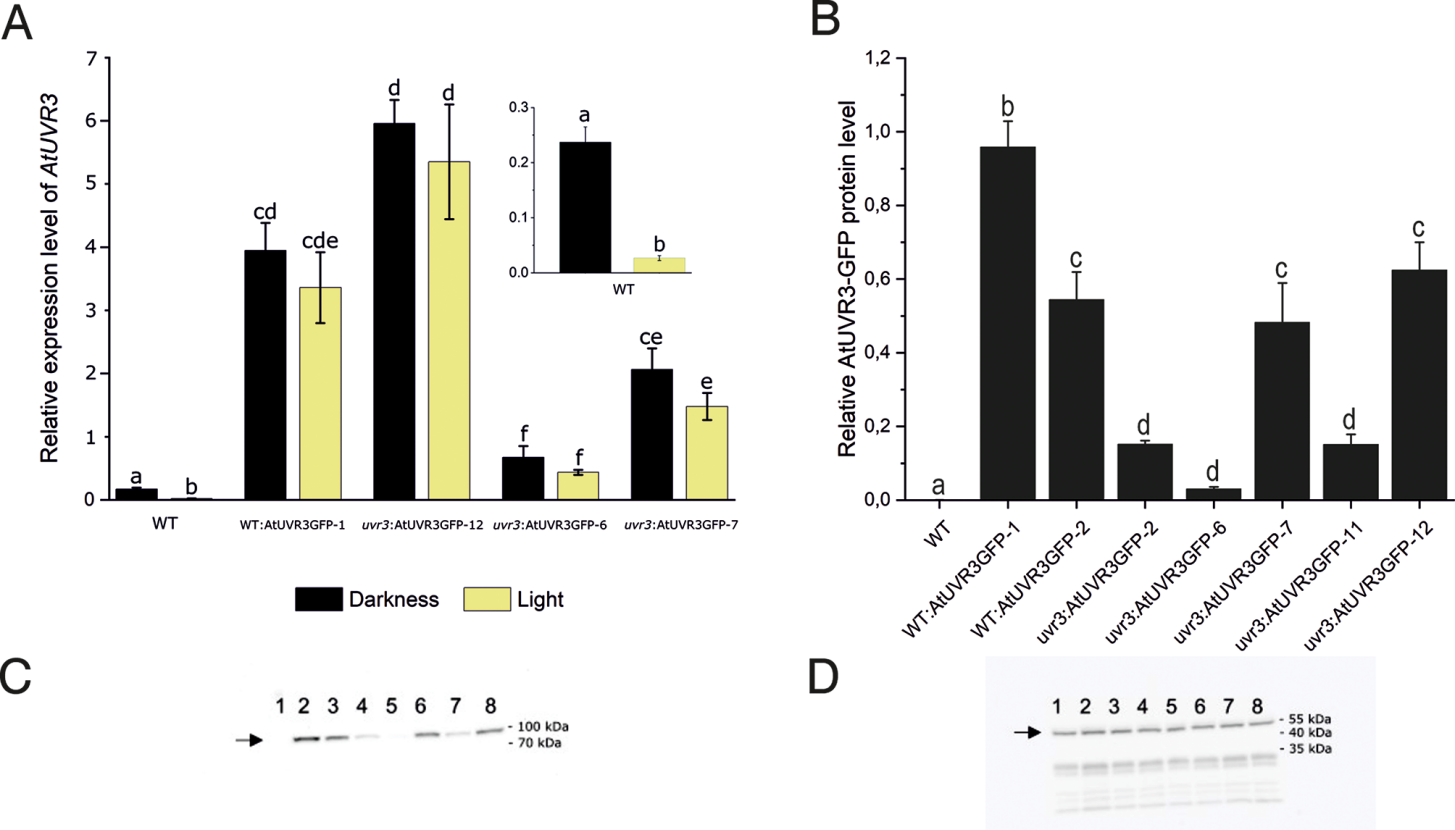
mRNA and protein levels of AtUVR3 in WT and transgenic Arabidopsis lines. **(A)** Light regulation of *AtUVR3* expression. Dark-adapted overnight 7-day-old seedlings of Arabidopsis WT and transgenic lines expressing *AtUVR3* under the control of 35S promoter were illuminated for 3 h in the culture chamber with white light (65–80 µmol·m^− 2^·s^− 1^) or left in darkness. Each bar corresponds to an average of three biological replicates. **(B)** The relative amount of AtUVR3 in dark-adapted overnight 7-day-old seedlings of Arabidopsis transgenic 35S: AtUVR3-GFP lines. Intensities of the chemiluminescent signal of the secondary antibodies against anti-GFP ones were normalized to actin levels in each sample. One of the three original, representative Western blot images (see also: Supplementary Fig. S1) used for densitometry analysis probed with **(C)** anti-GFP and **(D)** anti-actin antibodies. Proteins were extracted from 7 day-old Arabidopsis seedlings: (1) WT; (2) WT: AtUVR3GFP-1; (3) WT: AtUVR3GFP-2; (4) *uvr3*:AtUVR3GFP-2, (5) *uvr3*:AtUVR3GFP-6, (6) *uvr3*:AtUVR3GFP-7, (7) *uvr3*:AtUVR3GFP-11; (8) *uvr3*:AtUVR3GFP-12. Error bars in A and B show standard error. For any pair of bars, their means differ at the significance level of 0.05 if and only if they do not share a letter (tested with the Tukey method)

The real-time PCR analysis was used to characterize the transgenic *Arabidopsis* lines. The steady-state levels of *AtUVR3* transcripts were higher in Arabidopsis expressing *AtUVR3* under the control of the 35S promoter in the WT or *uvr3* mutant background than in WT plants (Fig. 1A). However, the differences between WT and transgenic Arabidopsis depended on the specific plant line tested. Compared to WT plants, the average levels of *AtUVR3* mRNA were over 4, 13, 25, and 38 times higher in dark-adapted *uvr3*:AtUVR3GFP-6, *uvr3*:AtUVR3GFP-7, WT: AtUVR3GFP-1 and *uvr3*:AtUVR3GFP-12 lines, respectively. Similar trends were observed between mRNA and protein levels of the transgene in the Arabidopsis lines (Fig. 1B, C and Supplementary Fig. S1). The plants with the highest levels of AtUVR3-GFP proteins i.e., WT: AtUVR3GFP-1 and *uvr3*:AtUVR3GFP-12, also had the highest levels of the transgene mRNA. Consequently, *uvr3*:AtUVR3GFP-6 plants, characterized by the lowest amount of AtUVR3-GFP protein, had the lowest level of *AtUVR3* mRNA. The light-regulation of the steady state levels of *AtUVR3* mRNA typical for WT plants was not preserved in the plants expressing the coding sequence (CDS) of *AtUVR3* gene under the 35S promoter. While white, blue, and red light strongly down-regulated *AtUVR3* expression in WT (as observed in previous studies, see: [38]), the mRNA levels in all transgenic lines tested remained unchanged under these light conditions (Fig. 1A and Supplementary Fig. S2).

### Photoreactivation of 6 − 4 PPs in nuclear DNA depends on the amount of AtUVR3

**Fig. 2:**
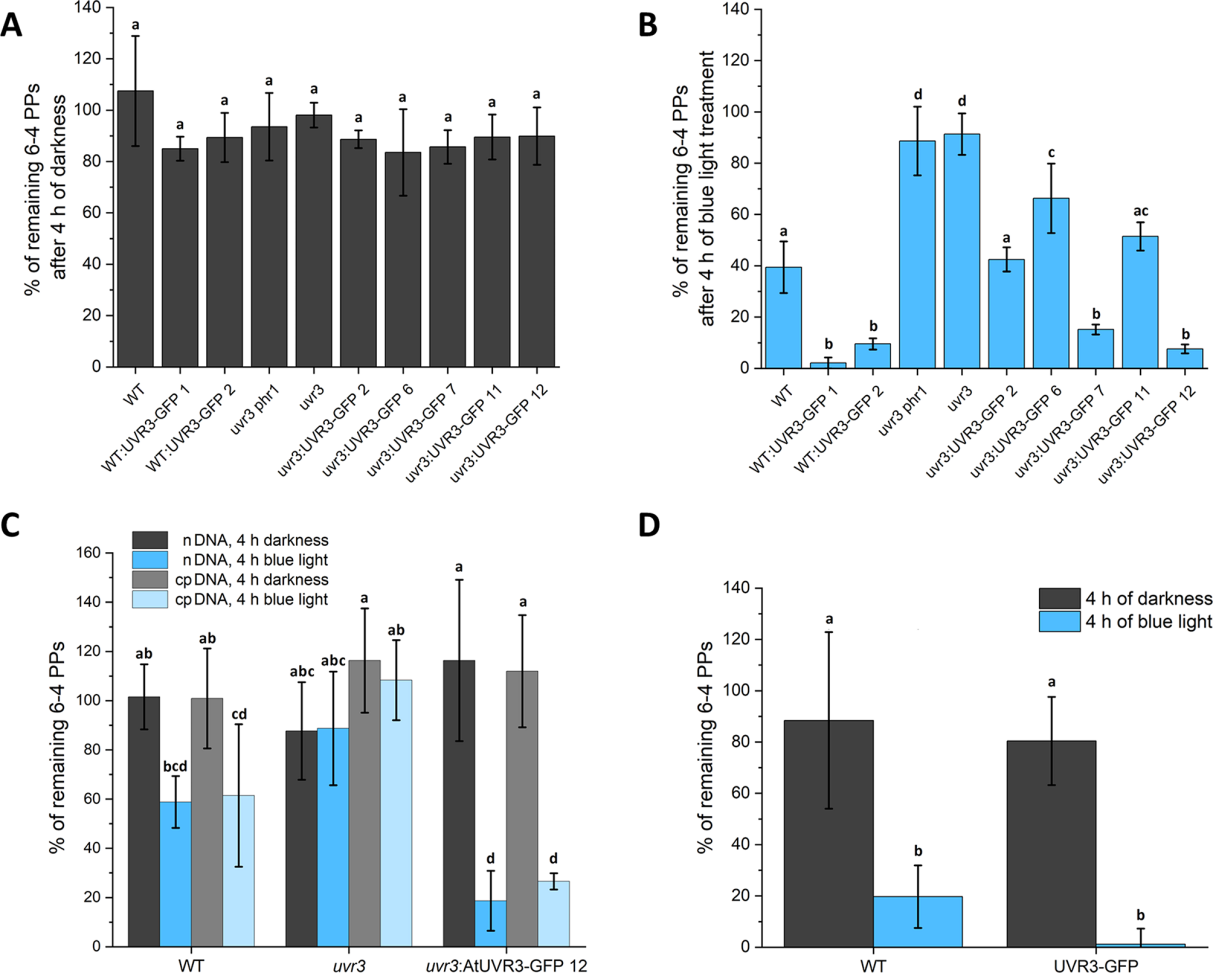
Photorepair of 6 − 4 PPs. The repair of 6 − 4 PPs in 7-day-old Arabidopsis seedlings after 4 h in **(A)** the darkness or **(B)** blue light. The removal of pyrimidine dimers in **(C)** cp. and nDNA isolated from leaves of Arabidopsis WT, *uvr3* mutant, and uvr3:AtUVR3GFP-12 and in **(D)** nDNA from leaves of *Nicotiana tabacum* WT and overexpressing AtUVR3-GFP. All plants were dark-adapted overnight and irradiated with UV-B (3.8 Wm^− 2^; 10 min). The percentage of 6 − 4 PPs is expressed as the ratio between the absorbances (450 nm) detected in the ELISA assay, measured for plants kept in darkness or under blue light (100 µmol·m^− 2^·s^− 1^) for 4 h after UV irradiation and those collected immediately after the UV treatment (i.e., 100%). Each bar corresponds to an average of four biological replicates. Error bars = SD. No statistically significant differences were found for data in panel A. In A, B, C, and D, the means of any two bars differ at the significance level of < 0.05 if and only if they do not share a letter (tested with the Tukey method, Supplementary Table S7)

To link the levels of AtUVR3 photolyase with the efficiency of 6 − 4 PP removal, we used the *uvr3* mutant, a set of transgenic lines expressing different levels of AtUVR3, and WT Arabidopsis plants (Fig. 1). Additionally, we compared the amounts of 6 − 4 PPs in the leaves of *Nicotiana tabacum* WT and AtUVR3-GFP plants. The levels of 6 − 4 PPs were assessed using an ELISA assay with anti 6 − 4 PP antibodies. qRT-PCR analysis with primers specific for chloroplast (qRT-RBCL_F/R), nuclear (qRT-AtRpoTp_F/R), and mitochondrial (qRT-RRN26_F/R) genomes was performed to determine the subfractions of these three genomes in the DNA used to measure 6 − 4 PP levels (Supplementary Tables S1 and S2). The total DNA consisted of approximately 97–99% nuclear DNA and 1–3% chloroplast DNA, with no mitochondrial DNA detected (Supplementary Table S3). Therefore, it is referred to as nuclear (n) DNA. In darkness, the removal of 6 − 4 PPs was similar in all Arabidopsis and *Nicotiana* lines tested (Fig. 2A, C, D). In *uvr3* and *uvr3phr1* mutants, the repair of 6 − 4 PPs was similar under both light and dark conditions, indicating a lack of photoreactivation (compare Fig. 2A, B). All Arabidopsis and *Nicotiana* transgenic lines exhibited more efficient blue light-dependent repair of 6 − 4 PPs compared to their parent lines (Fig. 2B compare lines: *uvr3*:AtUVR3GFP-2, *uvr3*:AtUVR3GFP-6, *uvr3*:AtUVR3GFP-7, *uvr3*:AtUVR3GFP-11, *uvr3*:AtUVR3GFP-12 vs. *uvr3*; WT: AtUVR3GFP-1 and WT: AtUVR3GFP-2 vs. WT; Fig. 2D - *Nicotiana* WT and AtUVR3-GFP). The most effective 6 − 4 PP photoreactivation was observed in both Arabidopsis transgenic lines with the WT background (i.e., WT: AtUVR3GFP-1/2) and two lines, progenies of the *uvr3* mutant transformed with AtUVR3-GFP under the control of the 35S promoter (i.e., *uvr3*:AtUVR3GFP-7/12). After 4 h of blue light irradiation, activating photolyase activity, about 40% of 6 − 4 PPs remained in WT Arabidopsis seedlings, whereas these lesions were hardly detected in the WT: AtUVR3GFP-1 line. Under the same conditions, about 10% of UV-B-induced 6 − 4 PPs were detected in the *uvr3*:AtUVR3GFP-12 line. In the parent *uvr3* line, over 90% of these lesions remained unrepaired under photoreactivating light. The results suggest that under the tested conditions the photoreactivation of 6 − 4 PPs in nDNA was more efficient in *N. tabacum* WT compared to Arabidopsis WT plants (Fig. 2B, D). After 4 h of blue light irradiation 20% and 40% of these lesions stayed unrepaired in tobacco and Arabidopsis leaves respectively. Similarly to Arabidopsis, overexpression of AtUVR3 resulted in a lower amount of unrepaired 6 − 4 PPs (almost at the detection limit) in stably transformed *N. tabacum* compared to its parent line (Fig. 2B, D). However, no statistical significance was found between the levels of unrepaired 6 − 4 PPs in WT and AtUVR3-GFP *N. tabacum* lines (Fig. 2D).

### 6 − 4 PPs are removed by AtUVR3 in Arabidopsis chloroplasts

To determine the photoreactivation of 6 − 4 PPs in chloroplasts, purification of these organelles was followed by DNA isolation. Using this approach, we obtained DNA consisting of over 95% chloroplast nucleoids, as measured by qRT-PCR (Supplementary Table S4).

One may consider if the 5% contamination by the nuclear genome significantly impacts the observed photoreactivation level. This can be estimated based on the likelihood of pyrimidine dimer formation in each genome. Approximately 14% of sequences in each genome i.e. nuclear (GenBank CP002684.1, CP002685.1, CP002686.1, CP002687.1, CP002688.1), chloroplast (GenBank: MK353213.1) and mitochondrial (EMBL Y08501.2, https://www.ebi.ac.uk/ena/browser/view/Y08501) consist of adjacent pyrimidines that can potentially form dimers under UV-B. In line with this, the amounts of 6 − 4 PPs in nDNA and cpDNA measured using the ELISA assay in the samples collected immediately after UV-B treatment were comparable implying the similar induction of these lesions in both genomes. Therefore, even if there was the preferential repair of nDNA lesions, its impact should not exceed a few percent.

The levels of 6 − 4 PPs were assessed using the ELISA assay in WT, *uvr3* and *uvr3*:AtUVR3GFP-12 (the overexpressor line showing high photolyase levels). The ratios between the 6 − 4 PP levels in DNA from chloroplasts isolated from samples collected immediately after UV-treatment and the levels after 4 h in darkness were similar in all lines (Fig. 2C). The photoreactivation of these DNA lesions in cpDNA was evident in WT but not in *uvr3* plants. Overexpression of AtUVR3 led to a very efficient removal of 6 − 4 PPs in cpDNA. Interestingly, in both WT and *uvr3*:AtUVR3GFP-12 leaves the levels of 6 − 4 PPs that remained unrepaired were comparable in nDNA and cpDNA.

### AtUVR3 is found in nuclei, mitochondria, and chloroplasts of transgenic *Arabidopsis thaliana* and *Nicotiana tabacum* plants

**Fig. 3:**
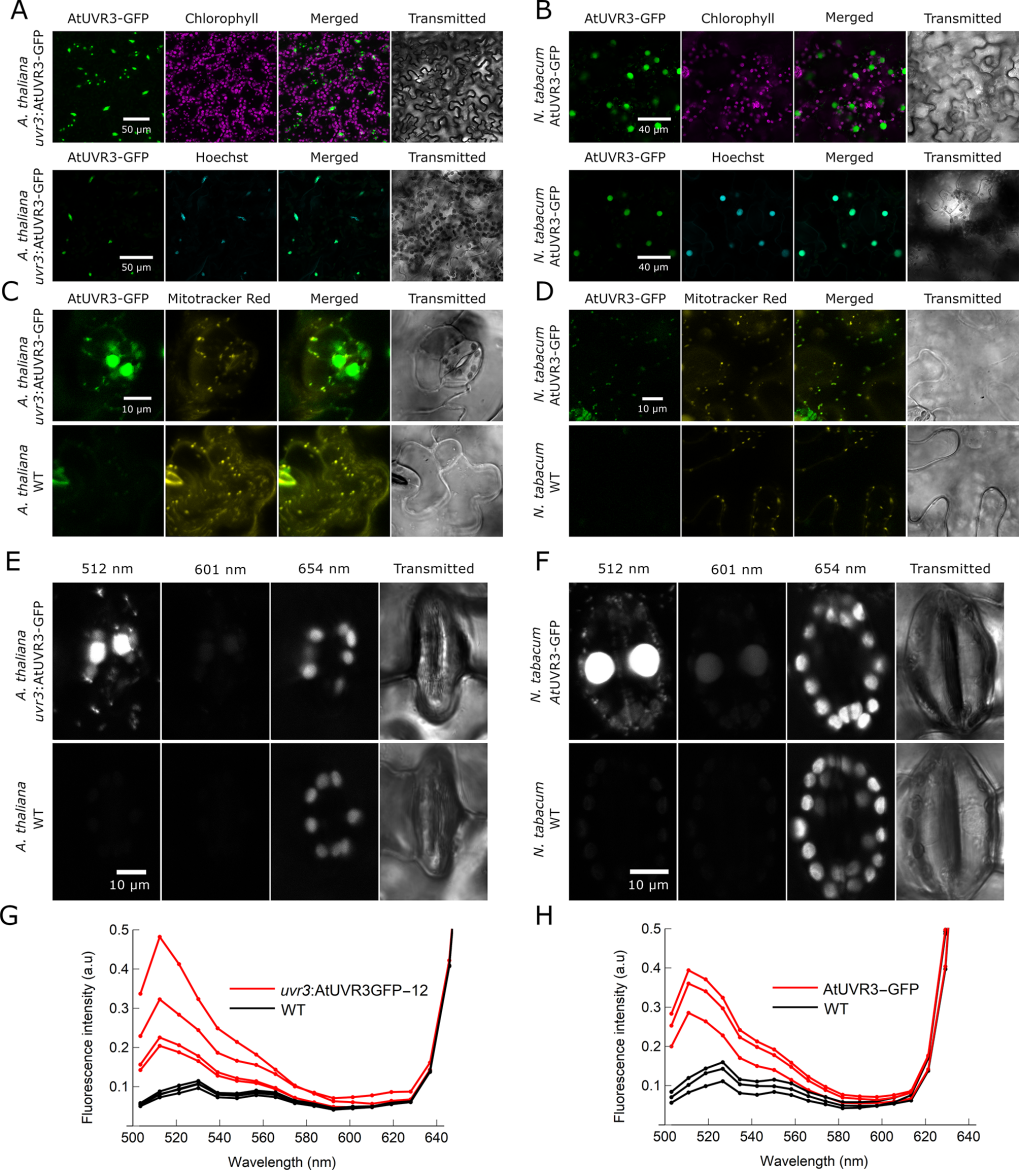
Subcellular localization of AtUVR3-GFP. **(A)** Localization of AtUVR3 in nuclei of the lower epidermal cells of transgenic Arabidopsis *uvr3*:AtUVR3-GFP-12 line. Maximum intensity projections were obtained from Z-stacks, recorded for ca. 40 μm, starting at the surface of the epidermis. Green fluorescence of chloroplasts in stable Arabidopsis lines is not visible at the detector gain settings adjusted to the high levels of GFP fluorescence in nuclei. Nuclear localization was confirmed in formaldehyde-fixed leaves with DNA-binding stain Hoechst 33,342 **(B)** Localization of AtUVR3 in nuclei of pavement cells of the lower epidermis of transgenic *Nicotiana tabacum* expressing AtUVR3-GFP. **(C)** Localization of AtUVR3 in mitochondria of pavement cells of the transgenic Arabidopsis *uvr3*:AtUVR3-GFP-7 line. Mitochondria were stained with MitoTracker™ Red. WT plants were used as an autofluorescence control **(D)** Localization of AtUVR3 in pavement cell mitochondria of transgenic *Nicotiana tabacum* expressing AtUVR3-GFP. **(E–H)** Localization of AtUVR3-GFP in chloroplasts of guard cells of the lower epidermis of rosette leaves of *A. thaliana uvr3*:AtUVR3GFP-12 plants (**E**), and the upper epidermis of leaves of transgenic *N. tabacum* plants expressing AtUVR3GFP (**F**). A set of 18 fluorescence channels of 8.9 nm width (λ stacks) was obtained, covering the range of 503–659 nm. The 488 nm laser line was used for excitation. WT plants were examined as autofluorescence controls. The channel centered at 512 nm shows fluorescence around the peak of GFP emission. In the channel centered at 654 nm, chlorophyll fluorescence is visible. Spectrum of chloroplast fluorescence were calculated from the λ stacks for *A. thaliana* (**G**) and *N. tabacum* (**H**). Each line represents the mean of approximately 15 stomatal apparatuses from a single plant. The values were normalized by the intensity of the 654 nm channel. For Arabidopsis, four transgenic and four WT plants were examined, for *Nicotiana*, three transgenic and three WT plants were examined

To investigate the localization of AtUVR3 the plants overexpressing AtUVR3-GFP under the 35S promoter, specifically one *N. tabacum* line and three independent Arabidopsis lines in the *uvr3* mutant background were used. Most GFP fluorescence was detected in the nuclei of epidermal and mesophyll cells of Arabidopsis and *Nicotiana tabacum* stably transformed with AtUVR3-GFP (Fig. 3A, B and Supplemental Figs. S3-S9). The nuclear localization of AtUVR3-GFP was confirmed with Hoechst 33342, a dye that binds double-stranded DNA (Supplemental Figs. S3 and S8). The nuclear shapes were typical for both species indicating that overexpression of AtUVR3 did not disturb nuclear morphology in our lines [40, 41]. Besides nuclei, green fluorescence was also observed in constantly moving structures in the Arabidopsis guard cell cytoplasm (Supplemental Movie S1). Similar structures were observed in the cytoplasm of pavement cells (Supplemental Fig. S9). Co-localization with MitoTracker™ Red, a mitochondrial dye, confirmed that GFP-fused AtUVR3 is transported to Arabidopsis and *N. tabacum* mitochondria (Fig. 3C, D). In both species, the green fluorescence was much weaker in chloroplasts than in nuclei and mitochondria. When excited with the 488 nm line, chloroplasts emit weak green fluorescence even in WT plants. To establish whether the green fluorescence in transgenic plants was from GFP or autofluorescence, we performed spectral imaging (λ-stacks) of chloroplasts in guard cells (Fig. 3E –H). The obtained coarse spectra show that the green fluorescence maximum in chloroplasts of AtUVR3-GFP-expressing plants is at approximately 512 nm, coinciding with the maximum of GFP fluorescence. In contrast, chloroplast fluorescence in WT plants exhibited a maximum in the green range at approximately 530 nm. Furthermore, the quantitative analysis of λ-stacks revealed that the chloroplast fluorescence in the green region was stronger in transgenic plants than in WT plants. These observations suggest that AtUVR3-GFP, in addition to nuclei and mitochondria, also localizes to chloroplasts in Arabidopsis and *N. tabacum*.

### AtUVR3 co-localizes with the plastid nucleoid in transiently transformed *N. benthamiana*

**Fig. 4:**
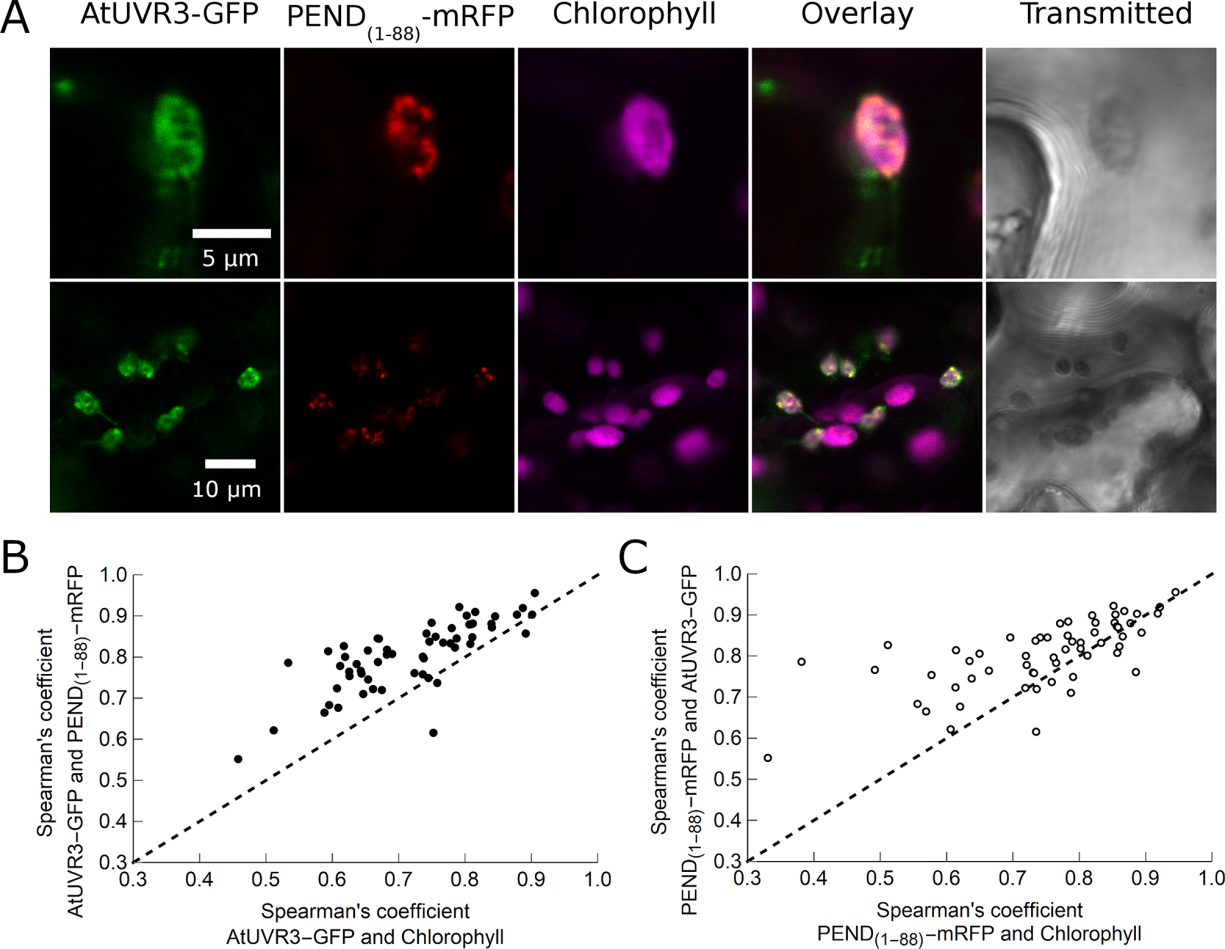
Co-localization of the chloroplast nucleoid marker PEND_(1–88)_-mRFP with AtUVR3-GFP. **(A)** Confocal images of *Nicotiana benthamiana* lower epidermis transiently transformed with PEND_(1–88)_-mRFP (red) and AtUVR3-GFP (green). Chlorophyll autofluorescence in magenta. Upper row: chloroplasts, lower row: an overview of the pavement cell. **(B)** Spearman’s correlation coefficient describing the strength of co-localization between AtUVR3-GFP and PEND_(1–88)_-mRFP, plotted against the correlation coefficient for AtUVR3-GFP and chlorophyll. **(C)** Spearman’s correlation coefficient for the PEND_(1–88)_-mRFP/AtUVR3-GFP pair plotted against the coefficient for PEND_(1–88)_-mRFP and chlorophyll. Each dot shows a pair of correlation coefficient values calculated for a single chloroplast. Dots above the dashed line correspond to chloroplasts in which AtUVR3-GFP and PEND_(1–88)_-mRFP were colocalized more with each other than with chlorophyll. The calculations were performed for 59 chloroplasts from 5 independent transformations

To gain insight into the (sub)chloroplastic localization of AtUVR3-GFP, co-transformation of this protein with PEND as the chloroplast nucleoid marker was performed. The red fluorescence of the first 88 amino acids of PEND fused with mRFP (PEND_(1–88)_-mRFP) [39] was observed in the nucleus and chloroplasts of transiently transformed *N. benthamiana* pavement cells (Fig. 4). In chloroplasts, PEND_(1–88)_-mRFP fluorescence appeared as speckles and more elongated regions (Fig. 4A). The green fluorescence of AtUVR3-GFP overlapped with the red fluorescence of PEND_(1–88)_-mRFP in chloroplasts (Fig. 4 and Supplemental Figs. S10, S11). AtUVR3-GFP often marked a greater area and its fluorescence was more diffused than that of PEND_(1–88)_-mRFP. However, the red fluorescence of PEND_(1–88)_-mRFP was always visible in the area occupied by AtUVR3-GFP. PEND and AtUVR3 colocalized more strongly with each other than with chlorophyll (Fig. 4B, C and Supplemental Figs. S10 and S11).

### Steady-state levels of AtUVR3 are not regulated by mutagens

To determine whether DNA lesions other than pyrimidine dimers influence the expression of *AtUVR3*, Arabidopsis WT seedlings were grown on media supplemented with different mutagenic agents: cisplatin (cisPt), mitomycin C (MitoC), bleomycin (Bleo), methylmethane sulfonate (MMS), zebularine (Zeb), and hydroxyurea (HU). The lowest concentrations of these mutagens, which were established in preliminary experiments to cause visible symptoms of seedling injury (such as inhibition of root growth, cotyledon and/or true leaf development), were used. None of the mutagens affected the mRNA levels of *AtUVR3* (Supplemental Fig. S12).

## Discussion

### AtUVR3 photolyase is responsible for photoreactivation of 6 − 4 PPs in Arabidopsis chloroplasts

A plethora of proteins responsible for the recognition of DNA lesions, their excision, DNA resynthesis using an undamaged DNA template, and finally its ligation, are required for dark repair pathways (for a review see: [7]). In contrast, photolyase is the single enzyme required for photoreactivation. Moreover, in this relatively fast process using only UVA/blue light energy, no template is needed as it simply reverses the reaction leading to pyrimidine dimer formation. Hence, in addition to the advantages mentioned above this repair pathway is also error-free. It is no surprise then that most CPDs and 6 − 4 PPs in plant DNA are removed via photoreactivation [2].

Data on photolyase functioning and the photoreactivation mechanism mainly come from experiments on bacteria or yeast cells or from in vitro studies on isolated proteins. *In planta* research on photolyases including their subcellular localization and the photoreactivation of organellar DNA is limited and focused mainly on CPDs. Rice OsPHR, a CPD-specific photolyase, has been found in nuclei, chloroplasts, and mitochondria [32, 42]. The photoreactivation of CPDs in these DNA-containing cellular compartments confirms the physiological role of the described OsPHR localization [32]. Arabidopsis CPD photolyase has been observed only in the nuclei of the transiently transformed Arabidopsis protoplasts [43]. Recently Dündar and co-workers [33] have described the photoreactivation of CPDs in mtDNA of WT Arabidopsis. This result, at least indirectly, indicates that the Arabidopsis CPD photolyase is transported both to nuclei and mitochondria and is active in these organelles.

No direct evidence showing the photoreactivation of 6 − 4 PPs in plant mitochondria or chloroplasts has been available yet. We observed light-dependent repair of these lesions in cpDNA of both WT and AtUVR3-GFP overexpressing plants in the *uvr3* mutant background (Fig. 2C). The photoreactivation of cpDNA from *uvr3*:AtUVR3-GFP-12 leaves was very efficient and comparable to the repair in nDNA. The removal of 6 − 4 PPs from WT cpDNA was less efficient but still similar to that observed in nDNA, significantly differing from repair without access to light. As expected the *uvr3* mutant showed no repair of 6 − 4 PPs in either chloroplast or nDNA regardless of light conditions after UV treatment. These results demonstrate that not only is the photoreactivation of 6 − 4 PPs active in plant chloroplasts but it is also the major mechanism responsible for the repair of these lesions in the chloroplasts of mature Arabidopsis leaves.

### Amount of AtUVR3 is crucial for the rate of 6 − 4 PP photoreactivation in nuclear and chloroplast DNA

Although photolyases need light for their activity, they bind to pyrimidine dimers even in the dark [44]. It has been calculated that in vivo about 90% of the yeast photolyase is bound to non-damaged DNA [45]. Short, millisecond flashes of photoreactivating light, which allow the repair of only one dimer per photolyase molecule, are used to determine the amount of these enzymes. Photoflash analysis indicates that the number of active CPD-specific photolyase molecules is 10–20 per cell in *E. coli*, and around 24,000 per cell in rice [46–48]. These results imply that these enzymes are not produced in excess when unneeded. Thus, in addition to the spectrum and intensity of light, the amount of photolyase molecules themselves seems to be a key limiting factor in photoreactivation.

Different kinetics of the repair of CPDs and 6 − 4 PPs in dark-adapted Arabidopsis plants irradiated with UV are widely known. A drop in the level of 6 − 4 PPs occurs shortly after turning on the photoreactivating light. In the case of CPDs, a clear delay in their decrease has been observed [36, 49], dependent on the length of the dark period preceding UV irradiation. The differences between the start of the repair of CPDs and 6 − 4 PPs are consistent with the opposite effect of light on the amounts of *AtPHR1* and *AtUVR3* transcripts. Whereas blue and red light up-regulate *AtPHR1*, these wavelengths down-regulate *AtUVR3* steady-state transcript levels [38, 50]. Assuming that the amount of photolyase molecules is reflected by their mRNA level in dark-adapted plants, the photoreactivation of 6 − 4 PPs can start immediately after turning on the photoreactivating light, but some time is needed to produce the CPD-specific photolyase. The significance of the photolyase amount on the course of photoreactivation has been confirmed by the more efficient removal of CPD dimers and the higher UV-resistance of rice and Arabidopsis plants overexpressing their native CPD photolyases [23, 43, 51].

The opposite effects of light on the expression of CPD and 6 − 4 PP specific Arabidopsis photolyases seem counterintuitive. The higher ratio of CPD formation under UV-B, along with higher sensitivity and mutation rates in plants lacking CPD but not 6 − 4 PP photolyase [14, 22] suggest a greater demand for CPD photolyase synthesis. The diurnal changes in UV irradiance and photosynthetically active radiation (PAR), including blue and red light that regulate CPD photolyase expression, may explain the low levels of CPD-specific photolyase in the morning. Under field conditions, its expression starts with sunrise when the UV-B to PAR ratio (and also the probability of CPD formation) is low and continues during the day when the UV-B: PAR ratio increases [17, 52]. The light-dependent decrease in mRNA levels of the *AtUVR3* gene, which encodes the 6 − 4 PP specific photolyase, depends on photosynthesis [38]. We hypothesize that plants do not produce new AtUVR3 proteins as long as photosynthesis and subsequent retrograde signals are undisturbed. In this case, these processes act as sensors of UV-B-induced plant injury, controlling AtUVR3 production.

Our results show not only more efficient 6 − 4 PP photoreactivation in plants overexpressing AtUVR3 compared with WT ones but also differences in 6 − 4 PP removal between transgenic Arabidopsis lines. The extent of this removal showed positive trends with *AtUVR3* mRNA and protein levels (compare Figs. 2 vs. 1). Photoreactivation was extremely effective in transgenic lines with the highest expression of AtUVR3, i.e., WT: AtUVR3GFP-1 and *uvr3*: AtUVR3GFP-12 in the WT and *uvr3* backgrounds, respectively. The same trend was observed for photoreactivation in chloroplasts. Although the level of AtUVR3-GFP in Arabidopsis chloroplasts was low compared to nuclei (Fig. 3), it was sufficient to effectively remove 6 − 4 PPs from cpDNA (Fig. 2C). This is additional evidence that although photolyases are not abundant proteins, the mechanism of photoreactivation with only one enzyme that may be used repeatedly, ensures the efficient removal of pyrimidine dimers.

### Regulation of the promoter activity is most likely the reason for the decrease of AtUVR3 transcript levels by light

In contrast to the CPD-specific photolyase encoded by *AtPHR1*, whose expression is up-regulated by light, the level of *AtUVR3* mRNA is reduced upon light treatment [26, 38, 53]. The light-dependent decrease of the *AtUVR3* mRNA level occurred in WT plants, but not in those expressing AtUVR3-GFP under the control of the 35S promoter (Fig. 1A and Supplemental Fig. S2). No regulation of *AtUVR3* mRNA levels was observed in all three independent transgenic Arabidopsis lines (*uvr3*:AtUVR3GFP-6, *uvr3*:AtUVR3GFP-7 and *uvr3*:AtUVR3GFP-12), including line 6, with *AtUVR3* expression comparable to dark-adapted WT plants. This rules out the possibility that an excess of *AtUVR3* mRNA clogged the degradation machinery. It seems that the light regulation of the steady-state levels of mRNA of this gene occurs due to changes in its promoter activity rather than the stability of its mRNA. However, the role of *AtUVR3* 5’-UTR and 3’-UTR, which are not present in the transgene, cannot be excluded.

### The mRNA levels of AtUVR3 are not regulated by DNA damaging agents

In contrast to the light, none of the mutagens tested in the study influenced *AtUVR3* transcript levels (Supplemental Fig. S12). DNA damage caused by various agents, including DNA cross-linking (cisPt and MitoC), induction of double-strand breaks (Bleo), base alkylation (MMS), inhibition of DNA methyltransferases (Zeb) and inhibition of DNA synthesis (HU), did not trigger a signal affecting *AtUVR3* expression. This is in contrast to the increase in yeast CPD photolyase observed upon treatment with alkylating agents MMS and N-methyl-N’-nitro-N-nitrosoguanidine (MNNG) as well as the UV mimetic agent 4-nitroquinoline-N-oxide (4-NQO), which leads to bulky purine adducts [53].

### AtUVR3 is transported mainly to nuclei when stably expressed in Arabidopsis and *Nicotiana tabacum*

We have previously shown that AtUVR3-GFP, a 6 − 4 PP-specific photolyase, is localized in nuclei, chloroplasts, and mitochondria of transiently transformed *N. benthamiana* epidermis and mesophyll cells [38]. In stably transformed 35S: AtUVR3-GFP lines of Arabidopsis and *N. tabacum* cv Samsun, GFP fluorescence was found mainly in nuclei (Fig. 3 and Supplemental Figs. S3-S8). It is worth noting that the fluorescence of both CPD and 6 − 4 PP photolyases (encoded by *AtPHR1* and *AtUVR3*, respectively) fused with GFP is mainly visible in nuclei of either transiently or stably transformed Arabidopsis plants ([43] Fig. 3 and Supplemental Figs. S3-S8). Thus, at least under the experimental conditions used, the majority of both photolyases are transported into nuclei when overexpressed in the native host.

Besides strongly labeled nuclei, weaker AtUVR3-GFP fluorescence was observed in the mitochondria of epidermal cells of transgenic Arabidopsis and *Nicotiana* (Fig. 3C, D and Supplementary Video S1). In these plants, the GFP fluorescence was also visible in guard cell chloroplasts. However, the chloroplast green fluorescence was relatively weak and did not form bright puncta, which were visible in chloroplasts of transiently transformed *N. benthamiana* (Fig. 3, compared with Supplemental Fig. S10 and [38]).

Differences between the subcellular localization of AtUVR3-GFP observed in transiently transformed *N. benthamiana* leaves with clearly labeled chloroplasts, and in stable Arabidopsis and *N. tabacum* lines, may be either species-specific or caused by the very high expression resulting from transient transformation. Furthermore, the distribution of AtUVR3 between cellular compartments may depend on the tissue, plant growth stage, or other factors. In this paper, the CDS of *AtUVR3* was expressed under the control of the 35S promoter, and thus the distribution of the native 6 − 4 PP photolyase in the cell may differ from the one described above. The subcellular localization may depend on the use of alternative promoters enabling transcription from different transcription start sites (TSS) or on alternative splicing [54]. The ATGpr software (https://atgpr.dbcls.jp/) predicts five TSSs in the canonical AtUVR3 (At3G15620.1) CDS. The annotation of the Arabidopsis Information Resource (TAIR) database proposes, in addition to the a canonical *AtUVR3 (At3G15620.1*) splicing variant, an alternative transcript (*At3G15620.2*) which results from differential splicing of the 11th exon. The proteins produced from these mRNAs share the first 436 amino acids and differ only at the C-terminal parts. The localization of AtUVR3 in chloroplasts depends on the C-terminal loop of the protein as truncated proteins either lacking the last 55 amino acids or with some C-terminal lysines mutated to arginines have not been found in these organelles. Moreover, the AtUVR3 proteins lacking the 120 amino acids from its N-terminus have been localized in chloroplasts [38]. Thus, the transcription from an alternative TSS, even if potentially changes the N-terminus of AtUVR3, should not influence its transport into chloroplasts, which depends on the C-terminal amino acids. Similarly, the alternatively spliced AtUVR3 protein seems to not be transported into chloroplasts. The expression level does not affect AtUVR3-GFP distribution in the cell, as a similar pattern, with the protein localized mainly in the nucleus was observed in Arabidopsis lines with different levels of this photolyase (Supplemental Figs. S3, S5, S6, S7).

The factors regulating the distribution of photolyases between the nucleus and organelles are unclear. The phosphorylation of the rice CPD photolyase is proposed to govern its partitioning between the nucleus, chloroplasts, and mitochondria, though the conditions promoting this phosphorylation are unknown [32]. Although no post-translational modifications of AtUVR3 have been described yet, it cannot be excluded that similar mechanism is active for this photolyase. As discussed above, the photoreactivation of 6 − 4 PPs strongly depends on the amount of AtUVR3. The levels of unrepaired 6 − 4 PPs in nDNA and cpDNA in WT plants after a 4-h blue light treatment are comparable, similar to those in *uvr3*:AtUVR3GFP-12 under the same conditions (Fig. 2C). This suggests that the distribution of AtUVR3 between the nucleus and chloroplasts in both plant lines is similar, with most of the protein localized in nuclei. To sum up, to the best of our knowledge, the subcellular localization of the native Arabidopsis 6 − 4 PP-specific photolyase appears to be similar to that reported here for the AtUVR3-GFP fusion protein.

Taking into account the sizes of chloroplast and nuclear genomes (see: *Materials and Methods* subsection: *Determination of mitochondrial*,* chloroplast and nuclear DNA*) and the variation in the number of copies of chloroplast nucleoids per cell, which ranges from 18 in mesophyll tissue to over 2,000 in mature leaves [55], one can calculate the molar ratio of chloroplast to nDNA in plant cells, ranging from 0.05 to 2.7. This relatively high fraction of cpDNA in the total cell DNA together with the low number of photolyases per plant cell and the limiting role of photolyase amount in pyrimidine dimer repair ([47], Figs. 1 and 2), underscores the presumably important role of photoreactivation in chloroplasts ([32], Fig. 2C). The fact that only a small fraction of photolyases is transported into these organelles (Fig. 3) makes the manipulation of photolyase levels a promising target for improving crop productivity under field conditions. Nevertheless, it cannot be excluded that pyrimidine dimers act as signaling molecules [24, 25] and, together with other UV-B-induced damages and subsequently activated signaling pathways, may prepare plants for environmental stresses. Recently it has been shown, that rice plants with high activity of CPD photolyase are more sensitive to rice blast fungus [56]. Will a similar effect be observed in plant responses to other abiotic or biotic stress conditions? Will a similar relationship occur in the case of plants overexpressing 6 − 4 PP specific photolyase? These questions are still waiting to be answered.

### AtUVR3 colocalizes with plastid nucleoids

Finally, the (sub)chloroplastic localization of AtUVR3 was analyzed. Our results show that AtUVR3 colocalizes with PEND in chloroplasts (Fig. 4). PEND is a plastid protein, postulated to anchor the nucleoid to the envelope in developing chloroplasts [39]. Proteins containing the first 88 amino acids from the PEND molecule, fused to a fluorescent tag, mark plastid nucleoids in different plant tissues [57]. Such engineered proteins are described to be transported exclusively to chloroplasts. However, we also observed red fluorescence of PEND_(1**–**88)_-mRFP in the nucleus (data not shown). The nuclear localization of PEND_(1**–**88)_-CFP has also been reported [58].

In the chloroplasts of *N. benthamiana*, we observed PEND_(1**–**88)_-mRFP localized in speckles and elongated regions (Fig. 4A). Similar fluorescence patterns have been described in chloroplasts from transiently transformed *N. tabacum* leaves, as well as cotyledons and leaves of transgenic Arabidopsis [57]. The co-localization of AtUVR3 with the chloroplast nucleoid labeled with PEND was visible in non-irradiated cells, thus without induction of 6 − 4 PPs (Fig. 4, Fig. S10 and Fig. S11). This is the first evidence suggesting an in vivo association of this photolyase with cpDNA. Interactions between photolyases and their substrates, i.e., pyrimidine dimers localized inside single or double-stranded DNA are widely studied. Isolated *E. coli* CPD-specific photolyase binds to undamaged plasmid DNA with an affinity about 7,500 times lower than to an oligomer containing a T < > T dimer [59]. Although yeast photolyase is associated with undamaged nDNA [45], little is known about the physiological role of such interactions. The movement of an isolated cyanobacterial photolyase over plasmid DNA was visualized with atomic force microscopy [60]. It was proposed, that also in vivo photolyases, although loosely bound to undamaged DNA, constantly slide over it to scan for pyrimidine dimers. Our results, although not definitive proof of the interaction of UVR3 photolyase with chloroplast nucleoid, may be a good starting point for testing this hypothesis.

## Materials and methods

### Plant material and culture conditions

Seeds of Arabidopsis: WT Columbia (N60000) and *uvr3* mutant (CS864134/WiscDsLox334H05) obtained from Nottingham Arabidopsis Stock Centre (Nottingham, UK), and *uvr3phr1* (WiscDsLox334H05 x WiscDsLox466C12) double mutant [61] were used for the experiments.

*Arabidopsis thaliana*, *Nicotiana benthamiana*, and *Nicotiana tabacum* cv Samsun were cultured in the soil as described in our previous study [38]. For the selection of transformed plants and UV-radiation experiments with seedlings, surface-sterilized seeds were sown in vitro on Gamborg (B5) medium with 1% sucrose and 0.7% agar. After 2 days of stratification at 4 °C, plants were cultured in a phytotron room (23 °C, with a 10 h light/14 h darkness photoperiod, 60–70 µmol·m^-2^·s^-1^ PPFD supplied by Sanyo FL40SS.W/37 lamps).

For mutagen and antimetabolite (HU) treatment, B5 media were supplemented with chemical compounds as indicated in Supplementary Table S5. MMS, cis-Pt, HU, and Zeb were obtained from Sigma Aldrich (St Louis, MO, USA). Bleo and MitoC were obtained from Selleckchem (UK). After stratification, plates were kept under incandescent white light of 100 µmol·m^-2^·s^-1^ (with a 16 h light/8 h darkness photoperiod) at 23 °C. Seedlings were collected on the tenth day of growth, 4 h after the onset of photoperiodic light. The material was frozen in liquid nitrogen immediately upon harvest and stored at -80 °C.

### Preparation of plasmids

The chloroplast nucleoid was labeled with the first 88 N-terminal amino acids of the plastid envelope DNA binding protein (PEND), fused at the C-terminus with mRFP. This construct is denoted as PEND_(1**–**88)_-mRFP. The genomic sequence including the fragment upstream of PEND 5’-UTR and its first 88 amino acids [57] was amplified using primers given in Supplementary Table S1, and cloned into the destination vector pSITEII-6C1 [62].

### Stable transformation of *Arabidopsis thaliana* and Nicotiana tabacum and transient transformation of *Nicotiana benthamiana*

For stable transformation, the *Agrobacterium* C58 strain carrying the pK7FWG2-AtUVR3 construct was used [38]. *Arabidopsis thaliana* (WT and *uvr3* mutants) and *Nicotiana tabacum* cv Samsun were transformed by the floral dip and leaf disc methods, respectively [63, 64]. The transformed plants were selected in vitro on media supplemented with kanamycin (50 mg/l). One *N. tabacum* and seven independent Arabidopsis lines (two in the WT background marked as WT:AtUVR3GFP-1/2 and five in the *uvr3* background marked as *uvr3*:AtUVR3GFP-2/6/7/11/12) were used for the experiments. Transient transformation was performed with the *Agrobacterium* C58 strain carrying a silencing inhibitor, P19. *N. benthamiana* transient transformation protocol is given in [65].

### Laser scanning confocal microscopy

*Staining.* To stain mitochondria, leaf pieces were syringe-infiltrated with either 170 nM solution of MitoTracker™ Red CMXRos (ThermoFisher Scientific) and incubated for 10 min (for *N. tabacum*), or a 100 nM CMXRos solution and incubated for 15 min (for *A. thaliana*). After incubation, the leaf pieces were rinsed several times with deionized water. To stain nuclei with Hoechst 33342, leaves were syringe-infiltrated with a fixative buffer (2% formaldehyde, 0.3% glutaraldehyde, 50 mM PIPES, pH 7.0, 10 mM EGTA, 5 mM MgSO_4_) and incubated for 1 h. The fixed leaves were then rinsed and stained for 1.5 h in Hoechst 33342 (5 µg/ml) dissolved in the same buffer used for fixation but without the aldehydes.

*Imaging microscopy.* The microscopy observations were performed using the AxioObserver.Z1 microscope, equipped with the LSM 880 confocal module (Carl Zeiss, Germany). The lower epidermis of transiently transformed *N. benthamiana* leaves was observed using a Plan-Apo 63×, 1.4 NA oil objective. The recording was performed in two separate tracks, which were switched on every line. In the first track, an argon-ion laser line of 488 nm was used for the excitation of GFP, while chlorophyll was excited with both the 488 nm line and the 633 nm He-Ne laser. Emission in the range 493–575 nm was recorded as the green channel, while emission in the 650–721 nm range was recorded as the magenta (chlorophyll) channel. In the second track, the 561 nm DPSS laser was used to excite mRFP with emission in the 588–650 nm range recorded as the red channel. For the quantitative assessment of the degree of co-localization in chloroplasts between AtUVR3-GFP, PEND_(1**–**88)_-mRFP, and chlorophyll, Spearman’s correlation coefficients were calculated using intensity in channels corresponding to different fluorophores. The calculations were performed in Mathematica 12.2 (Wolfram Research, USA).

Localization of AtUVR3 in the leaves of transgenic *A. thaliana* and *N. tabacum* plants was examined using a Plan-Neofluar 40×, 1.3 NA oil objective. An argon-ion laser line of 488 nm was used for the excitation of GFP and chlorophyll. When non-stained leaves were examined, the 493–598 nm and the 647–721 nm emission were recorded as the green (GFP) and the magenta (chlorophyll) channels, respectively. Channels were recorded in a single track. In experiments with MitoTracker™ Red (ThermoFisher) staining, the green (GFP, 494–544 nm), yellow (MitoTracker™, 597–623 nm), and magenta (chlorophyll, 664–735 nm) channels were recorded in a single track, with the 488 nm argon ion laser line and the 561 DPSS laser used for excitation. The fluorescence of leaves stained with Hoechst 3334 was recorded in three channels: cyan (426–479 nm, excitation with 405 nm line), green (499–525 nm, excitation with 488 nm line), and magenta (647–721 nm).

Spectral imaging (λ stacks) of the guard cells of transgenic Arabidopsis and *N. tabacum* leaves was performed in the 499–659 nm region, divided into 18 channels of 8.9 nm width. The Plan-Neofluar 40×, 1.3 NA oil objective was used. The 650–659 nm frames of the λ stacks were segmented using ImageJ to establish the boundaries of chloroplasts, while the 508–517 nm frames were used to delineate the nuclei. Binary images obtained with Otsu’s thresholding were used as the input to the Analyze Particles procedure in ImageJ. The contours of the obtained masks were then smoothed using the ShapeSmoothing plugin (https://imagej.net/plugins/shape-smoothing), subjected to the watershed algorithm to separate adjacent chloroplasts followed by another round of contour smoothing. In some cases, such masks were corrected manually to exclude mitochondria. The emission spectra were extracted from the areas within the boundaries of chloroplasts or nuclei using custom code written in Mathematica 12.2 (Wolfram Research, USA). Chloroplasts located within 2 μm of the edge of the nucleus were excluded from the spectrum to avoid accidental inclusion of scattered GFP emission from the nucleus. For WT *N. tabacum* plants, the outline of the nuclei was drawn manually on the transmitted light images.

Leaf cross-sections were prepared using the 7000smz-2 vibrating microtome (Campden Instruments, UK). Leaf pieces were infiltrated in PBS, embedded in 6% (w/v) low-melting point agarose (Agarose Super LM, Carl Roth), and cut using a ceramic blade (7550-1-SS, Campden Instruments).

### Measurement of AtUVR3 transcript levels

Nine-day-old in vitro grown Arabidopsis seedlings were used for real-time PCR experiments. After overnight dark adaptation the seedlings were either illuminated for 3 h with white (65–80 µmol·m^-2^·s^-1^, Philips MASTER TL-D 36 W/840), blue or red light (50 µmol·m^-2^·s^-1^, 470–660 nm respectively, LEDs) or kept in darkness for 3 h as a control (compare: [38]). The collected samples were immediately frozen in liquid nitrogen and stored at -80 °C. Total RNA was extracted using the Spectrum^™^ Plant Total RNA Kit (Sigma-Aldrich, St Louis, MO, USA) with an on-column DNA digestion step according to the manufacturer’s protocol. For reverse transcription 1,000 ng of RNA was used with the RevertAid M-MuLV Reverse Transcriptase Kit (Thermo Fisher Scientific, Vilnius, Lithuania) and oligo(dT)18 primers. Real-time PCR experiments were performed as described in [66], with all reactions run in triplicate. *PDF*,* SAND*, and *UBC* were used as reference genes [67]. The sequences of the primers used for qRT-PCR are given in Supplementary Table S1. For investigating the effect of mutagens *PDF* and *SAND* were used as reference genes. *SAND* and *UBC* genes were used in the experiment with blue and red light irradiation while *PDF*,* SAND*, and *UBC* were used to examine the impact of white light on *AtUVR3* expression.

### Determination of AtUVR3-GFP protein levels

Proteins were extracted from 7-day-old dark-adapted seedlings. Protein levels were determined as given in [68, 69] with some modifications. Samples were homogenized, weighed, and adjusted to equal mass. Whole proteins were extracted by homogenizing tissue in the following extraction buffer: 4% SDS, 2% (v/v) β-mercaptoethanol, 2 mM phenylmethylsulfonyl fluoride, and 0.1 M Tris-HCl, pH 8.5, followed by incubation at 80 °C for 3 min. The homogenate was centrifuged (16,000 g for 10 min at 4 °C), and the soluble fraction was used for immunoblot analysis. SDS–PAGE was performed on 12% polyacrylamide gels followed by subsequent semi-dry protein transfer (Bio-Rad). After the transfer, the membrane was blocked with 5% milk in phosphate-buffered saline (PBS) containing 0.5% Tween-20. Membranes were incubated with anti-GFP antibodies (sc-9996, Santa Cruz Biotechnology, Heidelberg, Germany) at a dilution of 1:250 overnight at 4 °C. Secondary antibodies (goat anti-mouse horseradish peroxidase-conjugated IgG, Sigma-Aldrich, St Louis, MO, USA), diluted 1:10,000, were applied at room temperature for 1 h. Signal detection was performed using a Clarity Western ECL Blotting Substrate (Bio-Rad, Hercules, CA, USA) with the BioSpectrum Imaging System (UVP, Analytic Jena US, USA). The intensities of the chemiluminescent signals were normalized to actin levels in each sample. For this, membranes were stripped using Restore Plus Western Blot Stripping Buffer (Thermo Fisher Scientific) and probed with anti-actin antibody (AS132640, Agrisera, Vännäs, Sweden), diluted 1:2500, at room temperature for 2 h, followed by secondary antibody (goat anti-rabbit horseradish peroxidase-conjugated IgG, Agrisera, Vännäs, Sweden) incubation and ECL detection. Densitometric quantification was performed using ImageJ.

### Chloroplast isolation

Chloroplasts for DNA damage measurements were isolated according to [70] with minor modifications. All steps were performed under a dim green light in a cold room, using precooled equipment and ice-cold buffers. For each isolation 2 g of leaves from 5-week-old soil-grown Arabidopsis were ground with a mortar and pestle in 6 ml of cold HSB buffer (1.25 M NaCl, 40 mM HEPES-KOH, pH 7.6, 2 mM EDTA, 0.1% (w/v) BSA, 0.1% β-mercaptoethanol). The homogenate was filtered through one layer of fleece and then through nylon meshes with 100 μm and 30 μm pores. The filtrate was centrifuged (5 min, 3,000 g, 4 °C), resuspended in the SDB buffer (0.33 M sorbitol, 100 mM HEPES-KOH, pH 7.6, 1 mM MgCl_2_, 2 mM EDTA, 0.1% (w/v) BSA). The resuspension was then loaded onto a step gradient consisting of 3 ml of 70% Percoll (Sigma-Aldrich) in SDB and 3 ml of 30% Percoll. Gradients were centrifuged (30 min, 1500 g, 4 °C), then the lower band, which contained intact chloroplasts, was collected using a glass Pasteur pipette and diluted with 13.5 ml of fresh SDB. The suspension was centrifuged three times (5 min, 3,000 g, 4 °C). After each centrifugation, the supernatant was discarded and the pellet was resuspended in 5 ml of fresh SDB. The final supernatant was discarded and the chloroplast pellet was frozen in liquid nitrogen and stored at -80 °C before DNA isolation.

### Measurement of pyrimidine dimer levels

*Light sources and measurements.* UV light was supplied using UV-B G8T5E (Ushio, Japan) tubes and filtered with UG-11 (Knight Optical, UK) and ZUS0325 (Asahi Spectra Co, Japan) filters additionally wrapped with two layers of cellulose acetate (95 μm thick). UV-B irradiance was measured with a probe (LP 471 UVB) of the HD2102.2 photo/radiometer (Delta OHM, Italy). Blue light irradiance was measured with the LI-190R sensor (Licor Biosciences, USA). Spectra were measured with a UV-VIS calibrated Black Comet C spectrometer (Stellar Net, USA) equipped with a cosine-corrector. The spectra of the light sources used in this work are shown in Supplemental Fig. S10.

For the measurement of pyrimidine dimer levels, in vitro cultured 7-day-old Arabidopsis seedlings, soil-grown 5-week-old Arabidopsis and 6-week-old *Nicotiana tabacum* were used. Plates with in vitro grown Arabidopsis seedlings or detached leaves placed flat on a wet paper towel were dark-adapted overnight, irradiated for 10 min with UV-B (3.8 W·m^-2^; for details see above) and collected immediately or after 4 h of either illumination with blue light (470 nm, 100 µmol·m^-2^·s^-1^) or dark adaptation. Control seedlings and leaves were kept in darkness without any irradiation. The samples were immediately frozen in liquid nitrogen and stored at -80 °C except those used for chloroplast isolation (see: *Chloroplast isolation* subsection of *Materials and Methods*). Each replicate used for isolation of total DNA consisted of: approximately 40 Arabidopsis seedlings, two halves of two separate tobacco leaves, or three leaves from 5-week-old Arabidopsis plants. An average of 30 leaves from 5-week-old Arabidopsis plants were needed to obtain 2 g of fresh mass for chloroplast isolation. Total DNA from Arabidopsis seedlings and *Nicotiana* leaves was isolated using the DNeasy Plant Mini Kit (Qiagen, Hilden, Germany). The DNA from adult Arabidopsis leaves and isolated chloroplasts were extracted using the Genomic Mini AX Plant (A&A Biotechnology, Gdańsk, Poland). DNA concentrations were measured using the PicoGreen™ assay (Invitrogen, Oregon, Canada). 6 − 4 PPs were quantified by ELISA assay using 64 M-2 (CosmoBio, Tokyo, Japan) antibodies [71]. The ELISA assay was performed according to the manufacturer’s protocol, with minor modifications. Briefly, 96-well polyvinylchloride flat-bottom microtiter plates (Greiner, Kremsmünster, Austria), pre-coated with 0.003% (w/v) protamine sulfate, were filled with 50 µl/well of heat-denatured DNA samples in PBS (0.1 ng/µl). The DNA was left overnight at 37 °C to completely dry. After each incubation step, each well was washed 4 times with PBS containing 0.05% Tween20. Microplates were incubated sequentially for 30 min at 37 °C with 0.5% skimmed milk in PBS, with the primary monoclonal antibody (64 M-2, diluted 1:2000 in PBS), with biotinylated F(ab)2 fragments of goat anti-mouse IgG (Invitrogen, Oregon, Canada, 1:4000 in PBS) and finally with HRP-conjugated streptavidin (R&D Systems, Minneapolis, USA, 1:200 in PBS). After the last incubation, each well was filled with 100 µl of the substrate (tetramethylbenzidine, stabilized with hydrogen peroxide, R&D Systems, Minneapolis, USA) and kept in darkness for 5 min. To stop the reaction, 1 M H_2_SO_4_ was added. Absorbance was measured at 450 nm with the TECAN microplate reader.

### Determination of mitochondrial, chloroplast and nuclear DNA

To establish the nuclear and organellar subfractions in the total DNA used for the determination of 6 − 4 PP levels, qRT-PCR analysis was performed. The sequences of the primers are given in Supplementary Table S1. The single-copy genes: ribosomal *RRN26*, chloroplast *RBCL* and nuclear *AtRpoTp* [72] were chosen for the analysis. The sizes of the mitochondrial, plastid, and nuclear genomes were assumed as 372 kb [73], 154 kb, and 115.4 Mb [74], respectively, to calculate the percentage of these subfractions in the total DNA.

### Statistical analysis

The statistical significance of the effects of irradiation conditions and the plant line (Figs. 1A and 4A) on the log-transformed *AtUVR3* expression level was assessed with a two-way factorial ANOVA with interaction, performed with the R software. The effect of the plant line on the AtUVR3 protein level, measured by Western blot densitometry (Fig. 1B), was assessed using one-way ANOVA on non-transformed data. The significance of the effects of plant line, DNA cellular origin (chloroplast/nuclear), and light treatment during reactivation (blue light/dark control treatment) on the amount of 6 − 4 PPs (Fig. 2B) was examined using three-way ANOVA with interaction. After significant ANOVA results, the differences in means of dependent variables between particular groups were examined using the Tukey method (packages emmeans and multcomp).

The amount of 6 − 4 PPs present in a sample was quantified by ELISA assay, conducted in triplicates. Seedlings from ten plant lines (WT, WT: AtUVR3-1, WT: AtUVR3-2, *uvr3phr1*, *uvr3*, *uvr3*:AtUVR3-2, *uvr3*:AtUVR3-6, *uvr3*:AtUVR3-7, *uvr3*:UVR3-11 and *uvr3*:UVR3-12) were subjected to initial UV irradiation. One-third of the seedlings were collected immediately after UV treatment, while the remaining plants were either kept in darkness or exposed to blue light, creating two different treatment conditions for the repair process of the primarily induced DNA lesions. The quotient of the 6 − 4 PP level after treatment and a baseline value was calculated for each sample. The experiment was performed in 5 independent repeats, except for the *uvr3*:AtUVR3-7 line, for which 4 repeats were performed.

The influence of plant line (ten levels) and different conditions during repair (two levels) on the remaining 6 − 4 PPs percentage was analyzed using a two-way ANOVA with interaction (Statistica v.13. software). The quotient of the 6 − 4 PP level was the dependent variable. Two “between-subject” factors were included: “plant line” with ten levels, and “repair conditions” with two levels. Statistical evaluation of the data showed that all effects: plant line, repair conditions, and the interaction between plant line and repair conditions were statistically significant (*p* < 0.01). The percentage of the remaining 6 − 4 PPs was significantly different among the various combinations of repair conditions and plant lines. Therefore, further exploration was performed using *post hoc* tests (Tukey HSD with unequal sample size, Supplementary Table S6). The ANOVA summary of all effects is given in Supplementary Table S7.

### Electronic supplementary material

Below is the link to the electronic supplementary material.

Supplementary Material 1

Supplementary Material 2

Supplementary Material 3

## Data Availability

The data underlying this article will be shared on reasonable request to the corresponding authors.

## Abbreviations

CPD: Cyclobutane pyrimidine dimer
cpDNA: chloroplast DNA
GFP: Green Fluorescent Protein
mtDNA: mitochondrial DNA
nDNA: nuclear DNA
PEND: Plastid envelope DNA binding protein
PHR1/UVR2: Photolyase1/UV resistance2
RFP: Red Fluorescent Protein
UVR3: UV repair defective 3
6 − 4 PPs: Pyrimidine 6 − 4 pyrimidones (6 − 4 photoproducts)
