# Supplementary material for: The photoreactivation of 6 − 4 photoproducts in chloroplast and nuclear DNA depends on the amount of the Arabidopsis UV repair defective 3 protein
Source: BMC Plant Biol. 2024 Jul 30;24:723. doi: 10.1186/s12870-024-05439-0 (PMC11287969; doi:10.1186/s12870-024-05439-0)
Supplement: Supplementary file 2 — Supplementary Material 2 [file 12870_2024_5439_MOESM2_ESM.pdf]

Supplementary materials

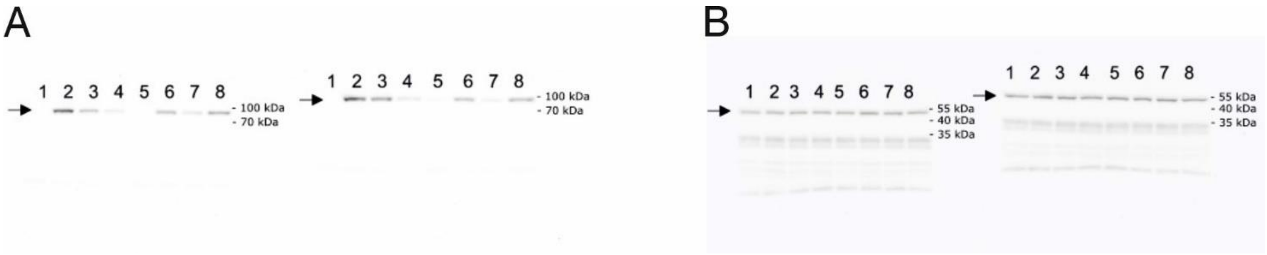

Fig. S1. Original images of Western blots used for densitometry analysis shown in **Fig. 1B** probed with **(A)** anti-GFP and **(B)** anti-actin antibodies. Proteins were extracted from 7 day-old *Arabidopsis* seedlings: (1) WT; (2) WT:AtUVR3GFP-1; (3) WT:AtUVR3GFP-2; (4) *uvr3*:AtUVR3GFP-2, (5) *uvr3*:AtUVR3GFP-6, (6) *uvr3*:AtUVR3GFP-7, (7) *uvr3*:AtUVR3GFP-11; (8) *uvr3*:AtUVR3GFP-12.

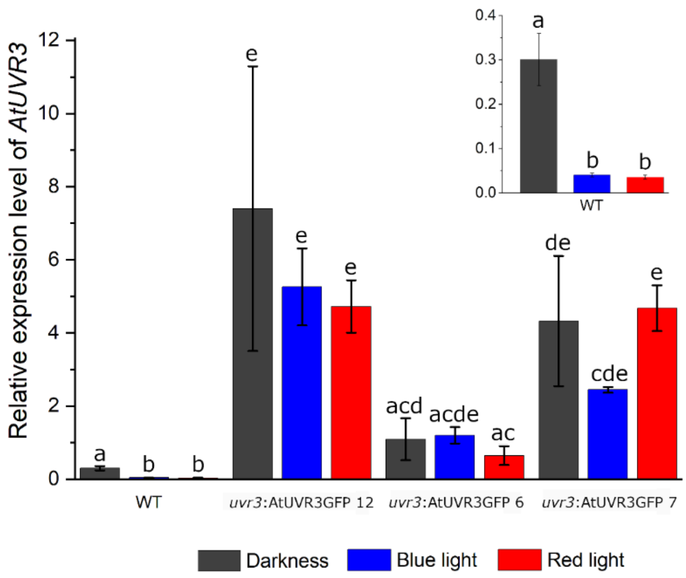

Fig. S2. Light regulation of *AtUVR3* expression. Dark-adapted overnight 7-day-old seedlings of Arabidopsis WT and transgenic *uvr3* lines expressing *AtUVR3* under the control of 35S promoter were illuminated for 3 h in the culture chamber with blue or red light (50 $\mu mol \cdot m^{-2} \cdot s^{-1}$) or left in darkness. Each bar corresponds to an average of three biological replicates. Error bars = SE. For any pair of bars, their means differ at the significance level of 0.05 if and only if they do not share a letter (tested with the Tukey method).

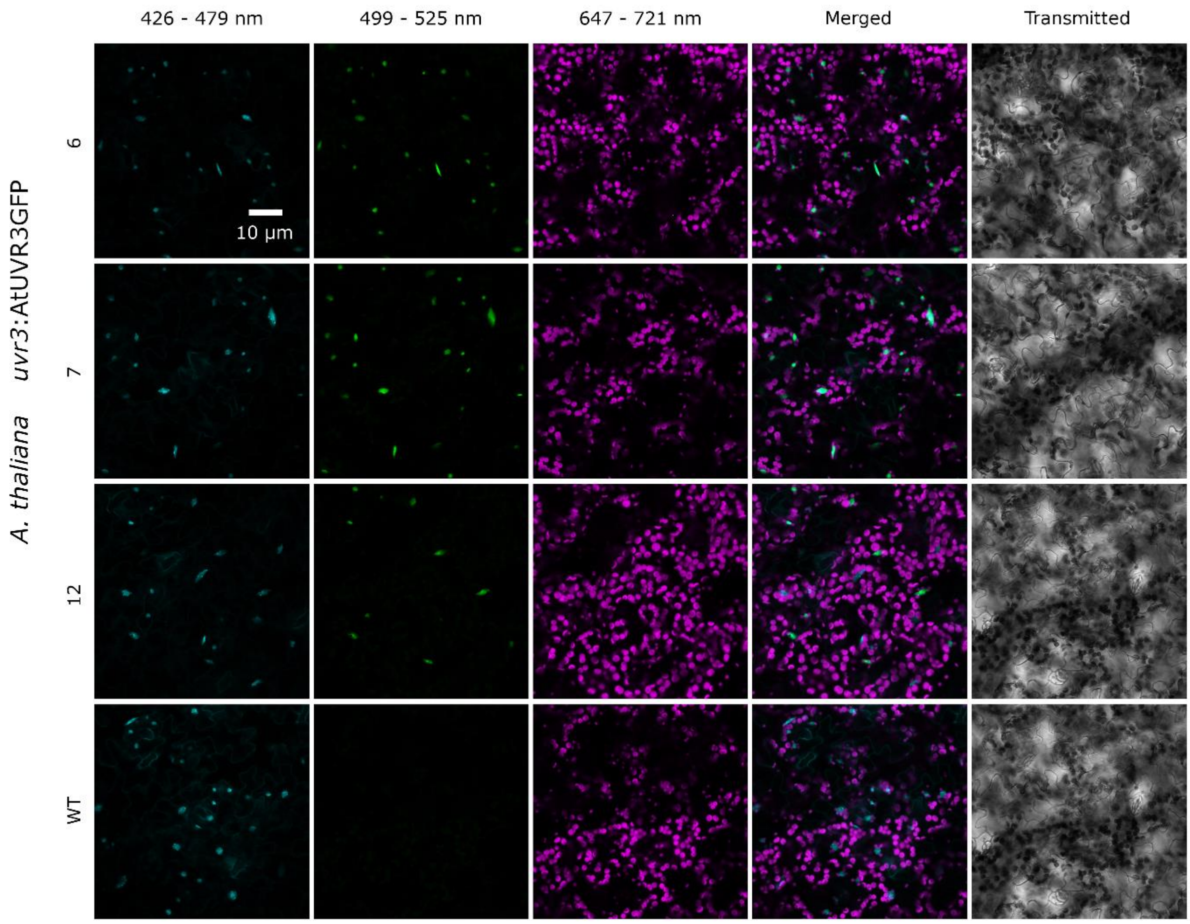

Fig. S3. Colocalization of nuclei stained with Hoechst and AtUVR3 in leaves of *Arabidopsis* WT and *uvr3*:AtUVR3GFP lines. Hoechst fluorescence (426-479 nm) in cyan, GFP fluorescence (499 – 525 nm) in green, chlorophyll autofluorescence (647 – 721 nm) in magenta.

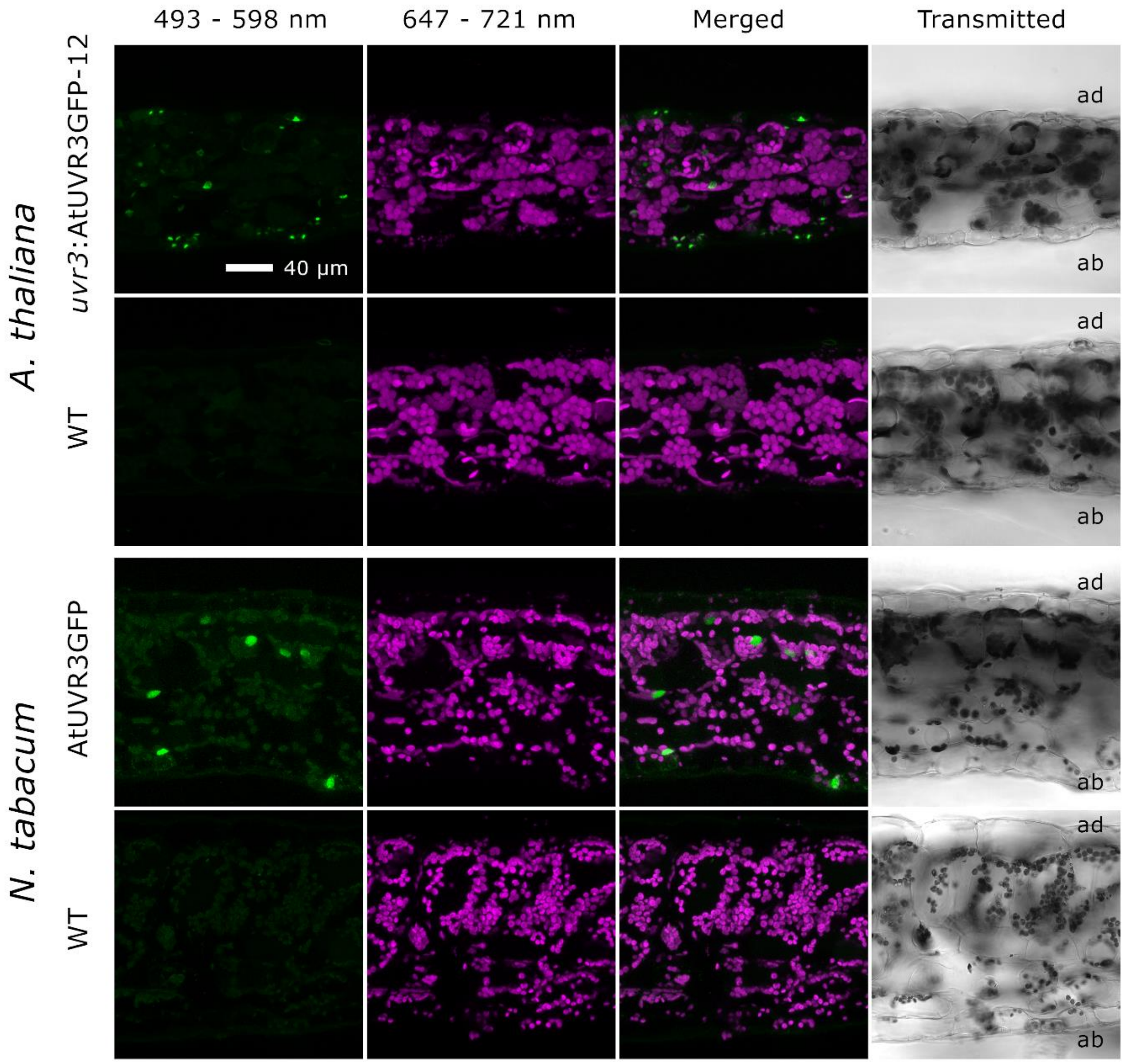

Fig. S4. Leaf cross-sections of leaves of *Arabidopsis uvr3*:AtUVR3GFP-12 and *Nicotiana tabacum* overexpressing AtUVR3-GFP. GFP fluorescence (493 – 598 nm) in green, chlorophyll autofluorescence (647 – 721 nm) in magenta. Ad – adaxial, ab – abaxial site of the leaf.

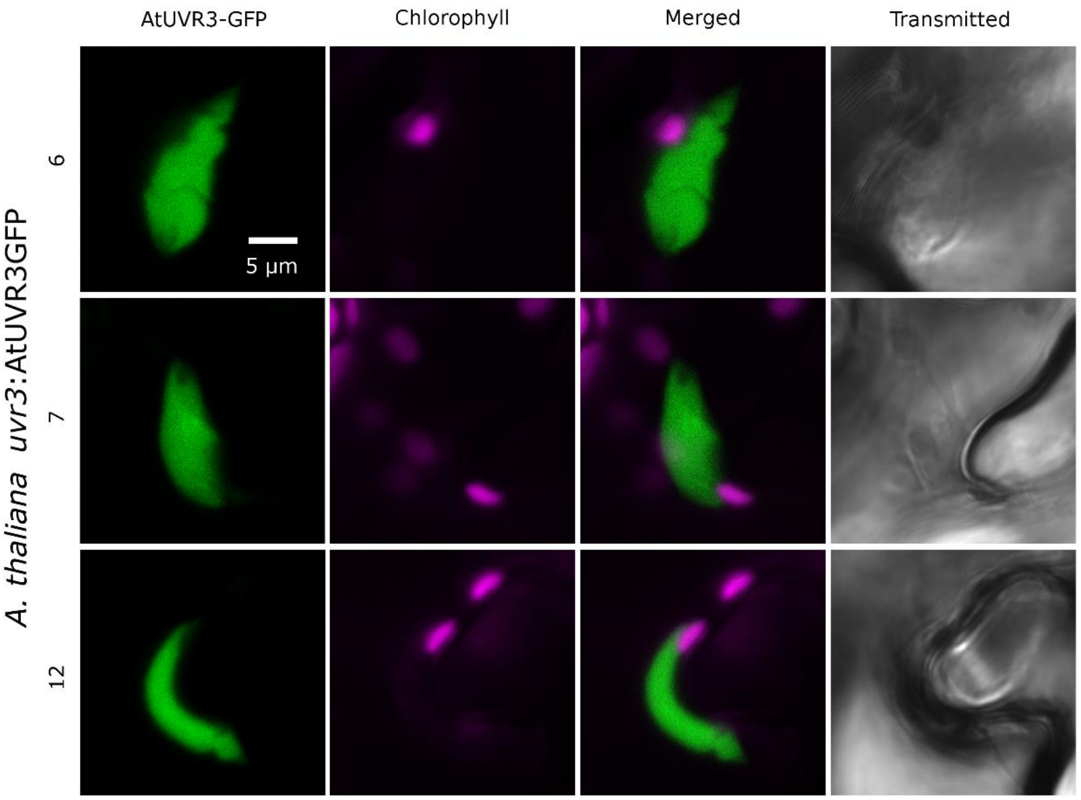

Fig. S5. AtUVR3 localization in the nuclei of pavement cells of the lower epidermis of *Arabidopsis uvr3*:AtUVR3GFP lines. GFP fluorescence (493 – 598 nm) in green, chlorophyll autofluorescence (647–721 nm) in magenta.

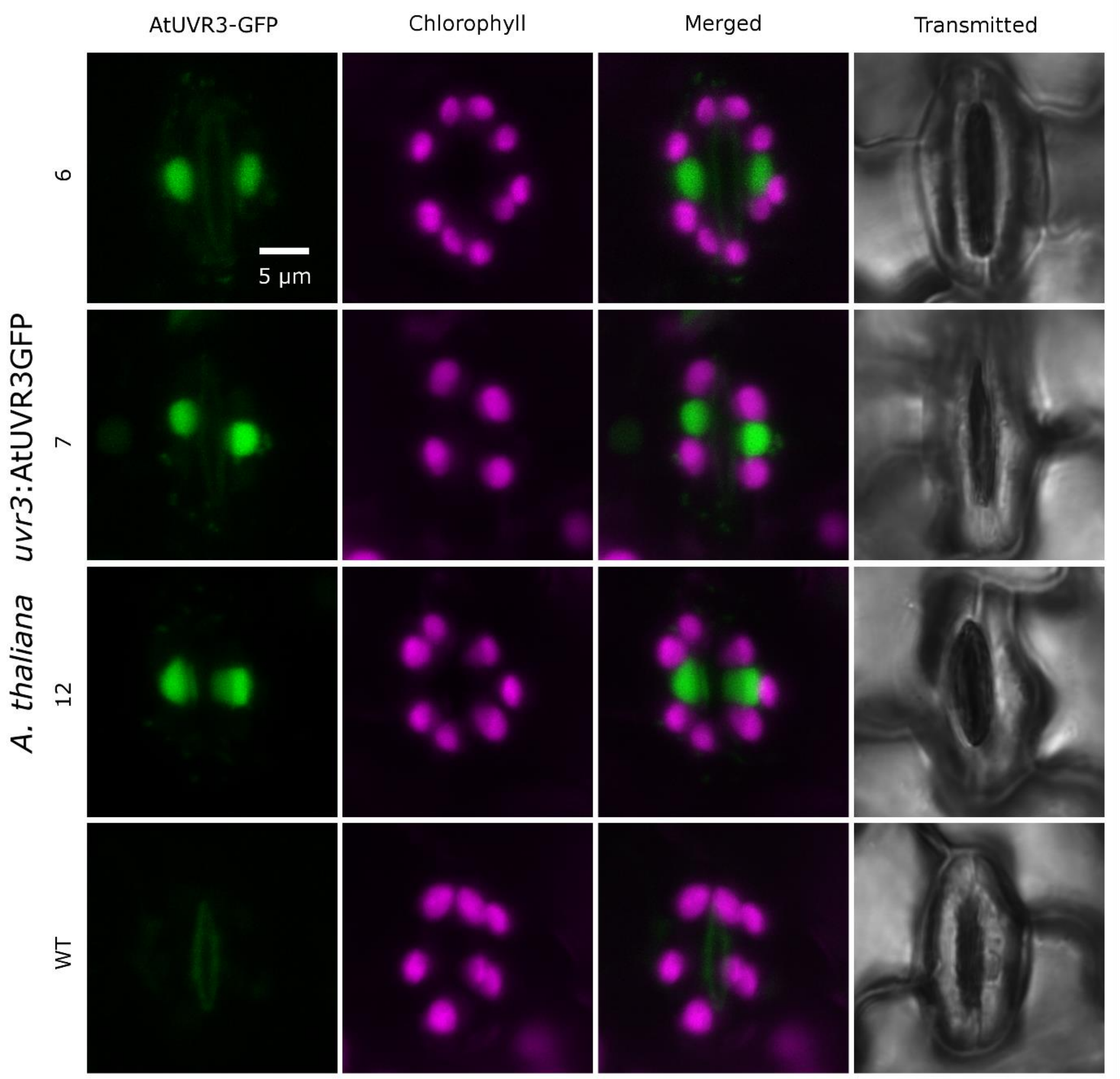

Fig. S6. AtUVR3 localization in nuclei of guard cells of the lower epidermis of *Arabidopsis uvr3*:AtUVR3GFP lines. GFP fluorescence (493 – 598 nm) in green, chlorophyll autofluorescence (647 – 721 nm) in magenta.

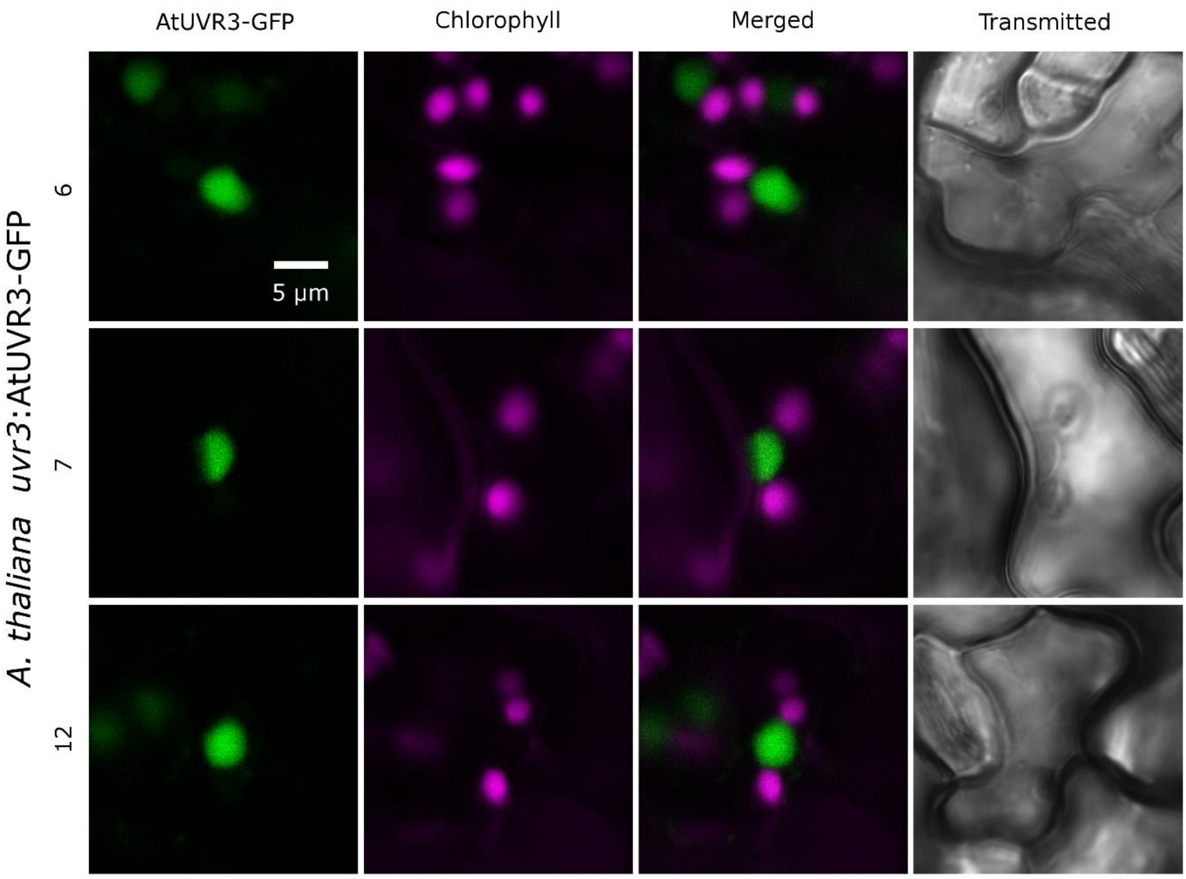

Fig. S7. AtUVR3 localization in nuclei of the subsidiary cells of the lower epidermis of *Arabidopsis uvr3*:AtUVR3GFP lines. GFP fluorescence (493 – 598 nm) in green, chlorophyll autofluorescence (647 – 721 nm) in magenta.

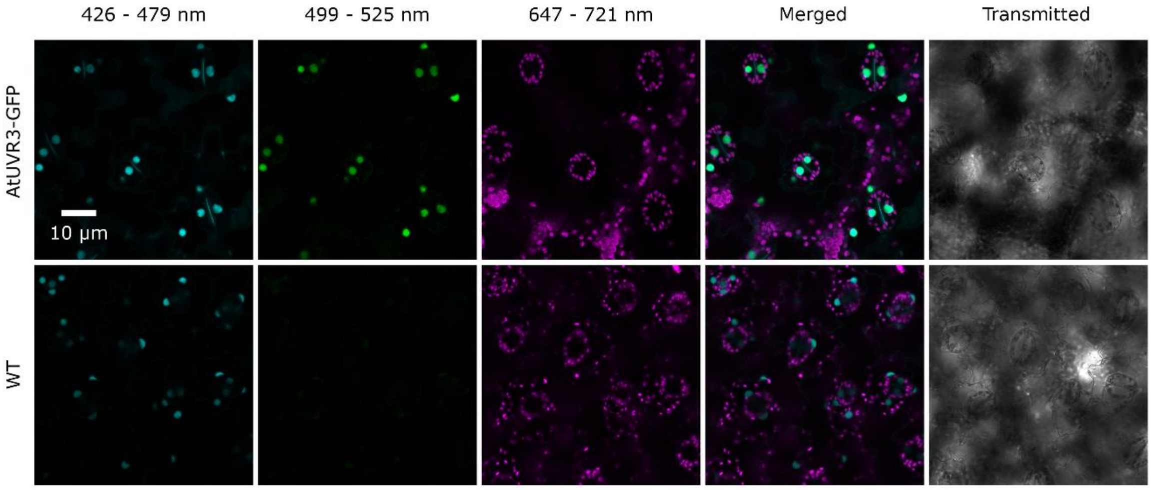

Fig. S8. Colocalization of nuclei stained with Hoechst and AtUVR3 in leaves of WT and transgenic *Nicotiana tabacum* overexpressing AtUVR3-GFP. Hoechst fluorescence (426-479 nm) in cyan, GFP fluorescence (499 – 525 nm) in green, chlorophyll autofluorescence (647 – 721 nm) in magenta.

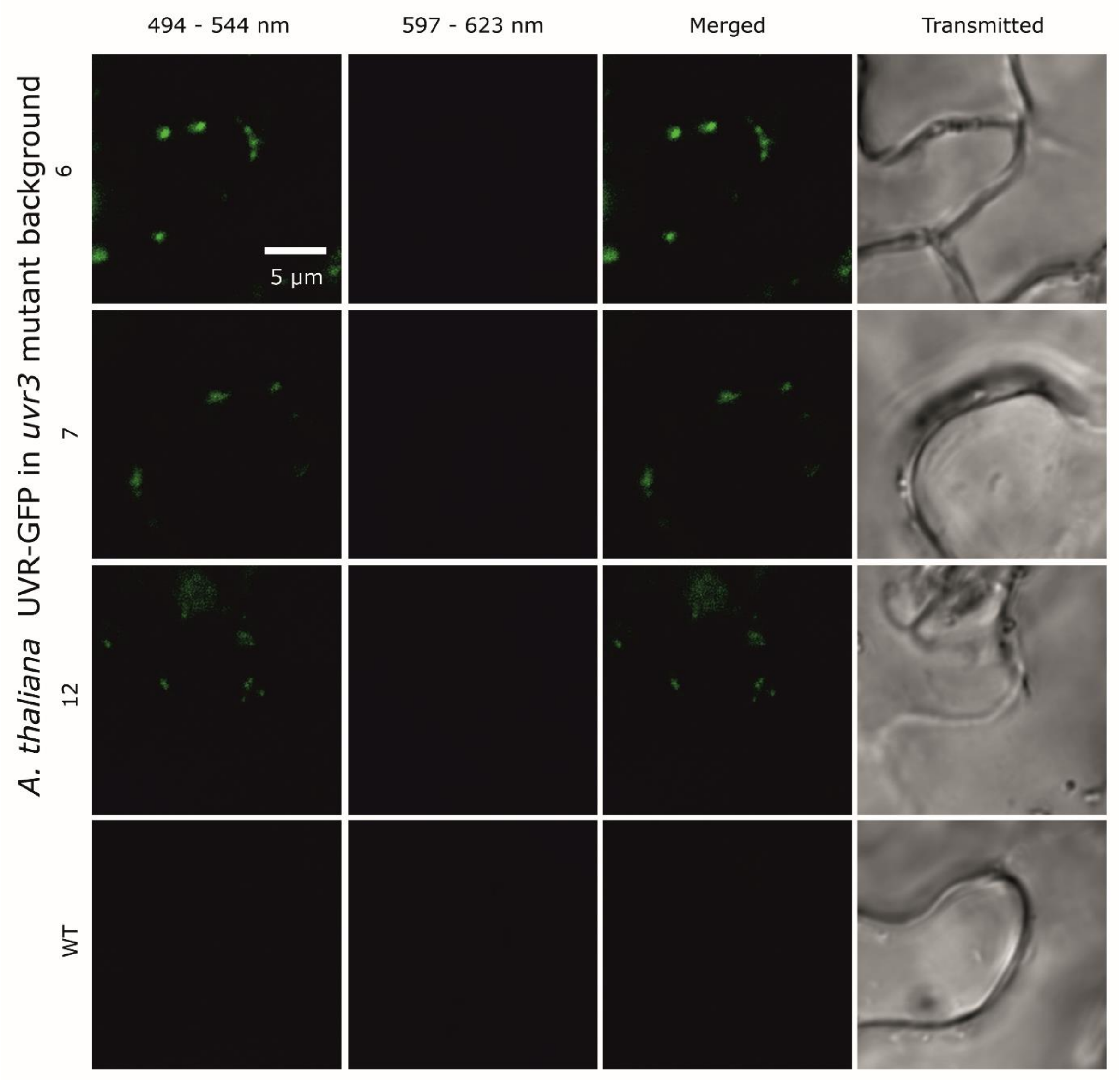

Fig. S9. Lower epidermis of water-infiltrated rosette leaves of *A. thaliana uvr3*:AtUVR3GFP and WT control. The confocal microscopy observations were performed on the same plant material and using the same settings as the observations of colocalization of GFP and MitoTracker Red fluorescence (Fig. 1 in the main body).

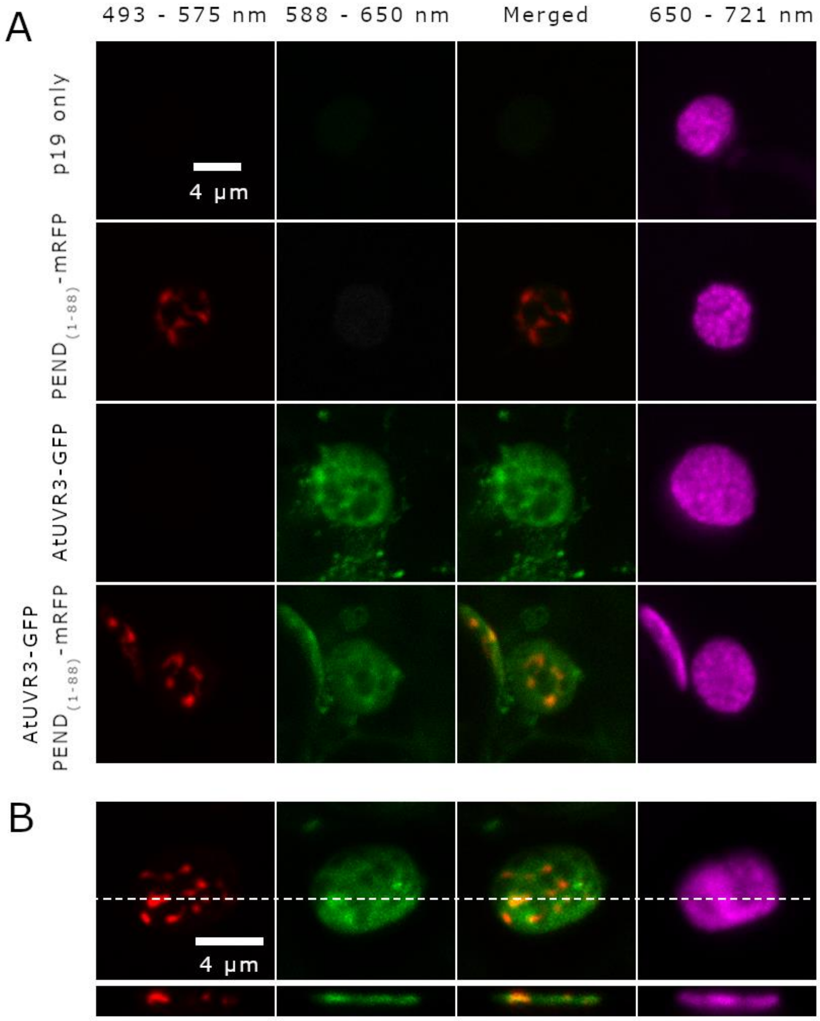

Fig. S10. Colocalization of AtUVR3-GFP and PEND-(1-88)-mRFP in chloroplasts of transiently transformed *N. benthamiana* leaves. (A) Projection images of chloroplasts of lower epidermis pavement cells of *N. benthamiana plants*. Leaves were infiltrated either only with bacteria carrying p19 silencing inhibitor (control, first row), or additionally with bacteria carrying vector for PEND-(1-88)-mRFP or AtUVR3-GFP expression, either separately (second and third row) or together (co-transformation, last row). (B) Projections and orthogonal cross-sections of Z-stacks of a chloroplast from a lower epidermis pavement cell of an N. benthamiana plant, expressing PEND-(1-88)-mRFP and AtUVR3-GFP. Cross-sections were recorded along dashed lines. Z-stacks were deconvolved using the Richardson-Lucy total variation algorithm implemented in the DeconvolutionLab2 (Sage et al. 2017) plugin of ImageJ. The point spread functions were recorded using beads of 100 µm diameter (TetraSpeck™ , Invitrogen). PEND(1-88)-mRFP in red, AtUVR3-GFP in green, chlorophyll autofluorescence in magenta.

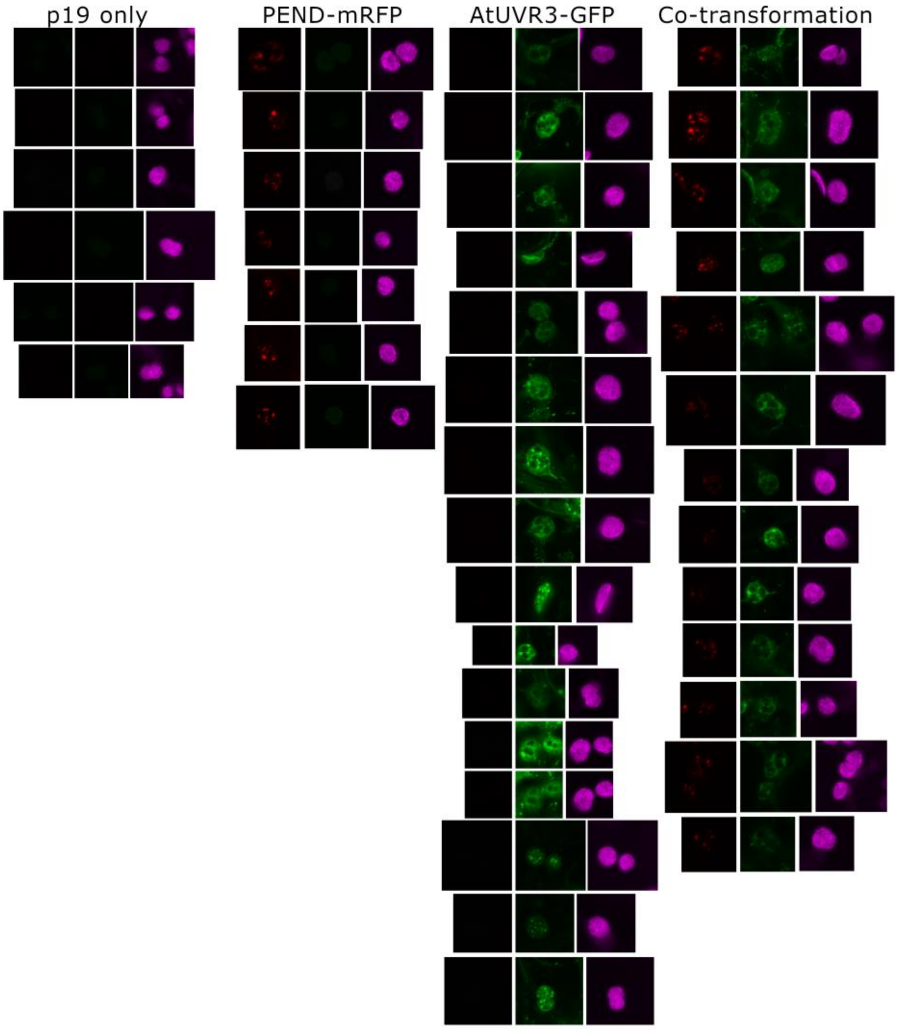

Fig. S11. The series of projection images of chloroplasts of *N. benthamiana* leaves transiently transformed with p19, PEND(1-88)-mRFP, AtUVR3-GFP or co-transformed with all three constructs. PEND(1-88)-mRFP in red, AtUVR3-GFP in green, chlorophyll autofluorescence in magenta.

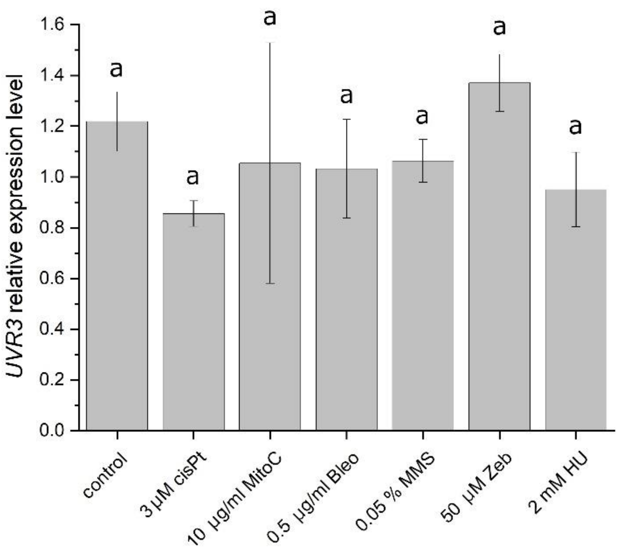

Fig. S12. Expression of *AtUVR3* in 10-day old Arabidopsis seedlings grown *in vitro* on either B5 media alone (control) or supplemented with mutagens. No statistical differences between means were found using Tukey's test.

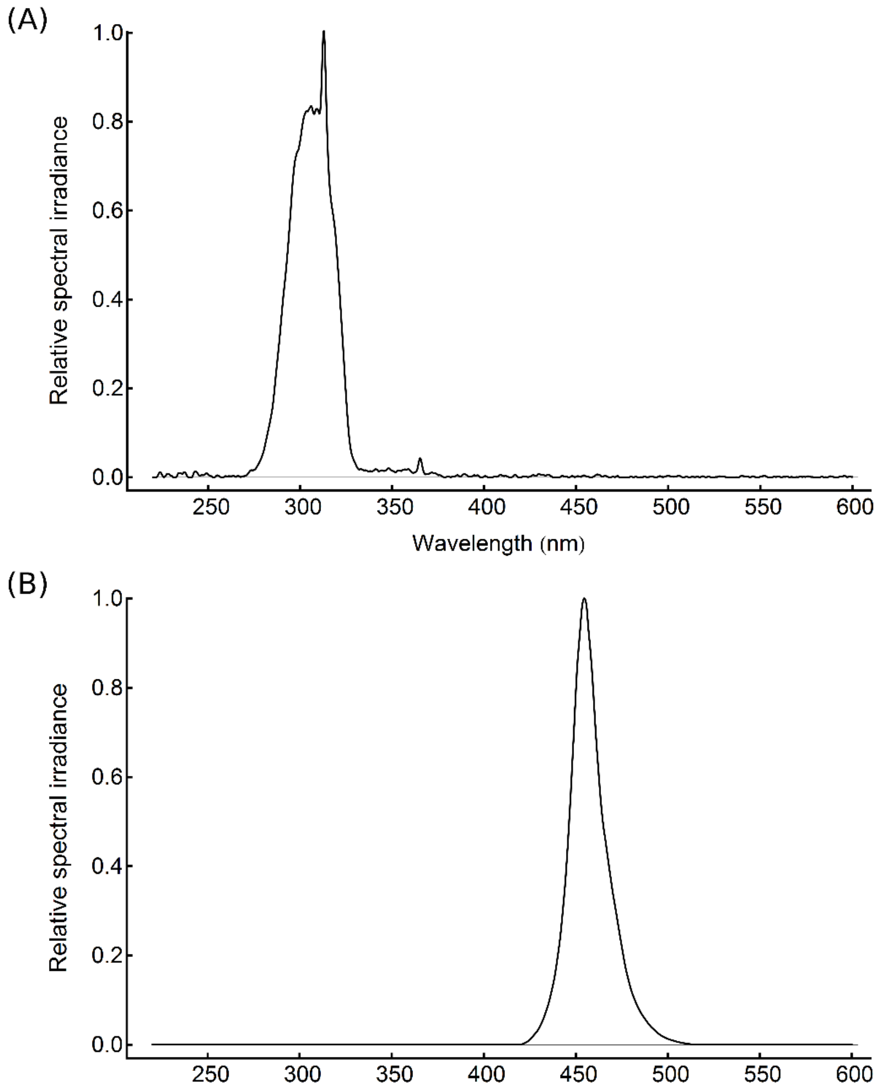

Fig. S13. (A) Spectrum of UV-B used in the experiments. Light emitted by USHIO UV-B G8T5E lamps was filtered through UG-11 (Knight Optical, UK), ZUS0325 (Asahi Spectra Co, Japan) filters and two layers of cellulose acetate (95 µm thick, Rachow Kunststoff-Folien, Germany). **(B)** Spectrum of blue light emitted by diodes (LXHL-PR09, Lumileds, Netherlands), which was used for the photoreactivation.

Table S1. The list of primers used for cloning of the first 88 aa of PEND and for real-time PCR. Lower case - gateway sequences.

Name	Primer sequence (5' - 3')
PEND_For	ggggacaagttgttacaaaaaagcaggcttcTCCTCAGA TGATAAGAATCACTCTTCG
PEND_Rev	ggggaccactttgtacaagaaagctgggtcACCAGGACC AAGGACTCTATTTTCTTGGAT
qRT-UVR3_F	TCCGGTTCTTGCTTGAGAGT
qRT-UVR3_R	CAAAGCAAAGCCTCTTCACC
qRT-UBC_F	CTGCGACTCAGGGAATCTTCTAA
qRT-UBC_R	TTGTGCCATTGAATTGAACCC
qRT-PDF2_F	TAACGTGGCCAAAATGATGC
qRT-PDF2_R	GTTCTCCACAACCGCTTGGT
qRT-SAND_F	AACTCTATGCAGCATTTGATCCACT
qRT-SAND_R	TGATTGCATATCTTTATCGCCATC
qRT-RBCL_F	TACCTGGTGTTCTGCCTGTG
qRT-RBCL_R	GCTACTCGGTTGGCTACGG
qRT-RRN26_F	AGCGGCTTAAGCCATTAGGTGTAG G
qRT-RRN26_R	ATTCGGAGTTTCCCTGGGGTTGG
qRT-AtRpoTp_F	CTGAATGCAGGTCGAAACTCGGG
qRT-AtRpoTp_R	GCTTGGAAGCCGTCTGCTAGAAC

According to Terasawa (2005) the *PEND* (At3g31570) forward primer is localized upstream of its 5'UTR.

Table S2. Efficiences of primer pairs used for calculation of DNA subfractions

Primer pair/genome	RT-qPCR primer efficiency [%]
qRT-RBCL_F/R (cp)	99
qRT-AtRpoTp_F/R (n)	99
qRT-RRN26_F/R (mt)	98

Table S3. Chloroplast (cp) and nuclear (n) subfractions in the total DNA.

Sample number	difference in (cp-nc) qRT-PCR cycle	cp:nc copy number ratio	% of nuclear DNA in total DNA	% of chloroplast DNA in total DNA
1	2.37	5.2	98.3	0.7
2	4.42	21.4	97.3	2.7
3	2.74	6.7	99.1	0.9

Table S4. Chloroplast (cp), mitochondrial (mt) and nuclear (n) subfractions in the DNA from isolated chloroplasts

Sample number	difference in (cp-n) qRT-PCR cycle	difference in (mt-n) qRT-PCR cycle	cp:n copy number ratio	mt:nc copy number ratio	% of nuclear DNA	% of mitochondrial DNA	% of chloroplast DNA
1	14.86	4.21	27603	19	2.64	0.16	97.20
2	13.90	3.68	14258	13	4.98	0.22	94.80

Table S5. List of mutagens used for *Arabidopsis* seedling *in vitro* culture

**Compound**	**Abbreviation**	**Solvent**	**Concentration**
Methyl methanesulfonate	MMS	water	0.003 %
Hydroxyurea	HU	water	2 mM
Mitomycin C	MitoC	water	10 µg mL^{-1}
Bleomycin	Bleo	DMSO	0.5 µg mL^{-1}
cis-Diammineplatinum(II) dichloride	Cis-Pt	DMSO	3 µM
Zebularine	Zeb	DMSO	40 µM

Table S6. The p-values obtained from Tukey's HSD pots hoc tests: (significant differences are indicated as red when <0.05)

class	Repair	Plant line	{1}	{2}	{3}	{4}	{5}	{6}	{7}	{8}	{9}	{10}	{11}	{12}	{13}	{14}	{15}	{16}	{17}	{18}	{19}	{20}
{1}	BL	WT		1.76E-04	3.49E-04	1.76E-04	1.76E-04	1.00E-00	1.52E-03	3.00E-02	8.27E-01	2.14E-04	1.76E-04	1.76E-04	1.76E-04	1.76E-04	1.76E-04	1.76E-04	1.76E-04	1.76E-04	1.76E-04	1.76E-04
{2}	BL	WT:UVR3-1	1.76E-04		9.99E-01	1.76E-04	1.76E-04	1.76E-04	1.76E-04	8.63E-01	1.76E-04	1.00E-00	1.76E-04	1.76E-04	1.76E-04	1.76E-04	1.76E-04	1.76E-04	1.76E-04	1.76E-04	1.76E-04	1.76E-04
{3}	BL	WT:UVR3-2	3.49E-04	9.99E-01		1.76E-04	1.76E-04	1.85E-04	1.76E-04	1.00E-00	1.76E-04	1.00E-00	1.76E-04	1.76E-04	1.76E-04	1.76E-04	1.76E-04	1.76E-04	1.76E-04	1.76E-04	1.76E-04	1.76E-04
{4}	BL	uvr3phr1	1.76E-04	1.76E-04	1.76E-04		1.00E-00	1.76E-04	2.10E-02	1.76E-04	1.77E-04	1.76E-04	9.87E-01	1.00E-00	1.00E+00	1.00E-00	9.78E-01	1.00E+00	1.00E-00	1.00E-00	1.00E+00	1.00E+00
{5}	BL	uvr3	1.76E-04	1.76E-04	1.76E-04	1.00E-00		1.76E-04	3.33E-03	1.76E-04	1.76E-04	1.76E-04	1.00E-00	1.00E-00	1.00E-00	1.00E-00	1.00E-00	1.00E-00	9.95E-01	1.00E-00	1.00E-00	1.00E-00
{6}	BL	uvr3:UVR3-2	1.00E-00	1.76E-04	1.85E-04	1.76E-04	1.76E-04		1.33E-02	4.62E-03	9.94E-01	1.78E-04	1.76E-04	1.76E-04	1.76E-04	1.76E-04	1.76E-04	1.76E-04	1.76E-04	1.76E-04	1.76E-04	1.76E-04
{7}	BL	uvr3:UVR3-6	1.52E-03	1.76E-04	1.76E-04	2.10E-02	3.33E-03	1.33E-02		1.76E-04	4.94E-01	1.76E-04	5.03E-04	1.26E-01	1.40E-02	1.19E-03	2.14E-04	6.97E-02	2.28E-01	2.20E-01	1.26E-02	1.03E-02
{8}	BL	uvr3:UVR3-7	3.00E-02	8.63E-01	1.00E-00	1.76E-04	1.76E-04	4.62E-03	1.76E-04		1.99E-04	1.00E-00	1.76E-04	1.76E-04	1.76E-04	1.76E-04	1.76E-04	1.76E-04	1.76E-04	1.76E-04	1.76E-04	1.76E-04
{9}	BL	uvr3:UVR3-11	8.27E-01	1.76E-04	1.76E-04	1.77E-04	1.76E-04	9.94E-01	4.94E-01	1.99E-04		1.76E-04	1.76E-04	1.86E-04	1.76E-04	1.76E-04	1.76E-04	1.88E-04	2.07E-04	2.71E-04	1.76E-04	1.76E-04
{10}	BL	uvr3:UVR3-12	2.14E-04	1.00E-00	1.00E-00	1.76E-04	1.76E-04	1.78E-04	1.76E-04	1.00E-00	1.76E-04		1.76E-04	1.76E-04	1.76E-04	1.76E-04	1.76E-04	1.76E-04	1.76E-04	1.76E-04	1.76E-04	1.76E-04
{11}	D	WT	1.76E-04	1.76E-04	1.76E-04	9.87E-01	1.00E-00	1.76E-04	5.03E-04	1.76E-04	1.76E-04	1.76E-04		8.09E-01	9.94E-01	1.00E-00	1.00E+00	9.87E-01	6.58E-01	8.65E-01	9.95E-01	9.97E-01
{12}	D	WT:UVR3-1	1.76E-04	1.76E-04	1.76E-04	1.00E-00	1.00E-00	1.76E-04	1.26E-01	1.76E-04	1.86E-04	1.76E-04	8.09E-01		1.00E-00	9.92E-01	7.17E-01	1.00E-00	1.00E-00	1.00E+00	1.00E-00	1.00E-00
{13}	D	WT:UVR3-2	1.76E-04	1.76E-04	1.76E-04	1.00E+00	1.00E-00	1.76E-04	1.40E-02	1.76E-04	1.76E-04	1.76E-04	9.94E-01	1.00E-00		1.00E-00	9.90E-01	1.00E+00	1.00E-00	1.00E-00	1.00E+00	1.00E+00
{14}	D	uvr3phr1	1.76E-04	1.76E-04	1.76E-04	1.00E-00	1.00E-00	1.76E-04	1.19E-03	1.76E-04	1.76E-04	1.76E-04	1.00E-00	9.92E-01	1.00E-00		1.00E-00	1.00E-00	9.60E-01	9.99E-01	1.00E-00	1.00E-00
{15}	D	uvr3	1.76E-04	1.76E-04	1.76E-04	9.78E-01	1.00E-00	1.76E-04	2.14E-04	1.76E-04	1.76E-04	1.76E-04	1.00E+00	7.17E-01	9.90E-01	1.00E-00		9.94E-01	5.31E-01	9.06E-01	9.93E-01	9.95E-01
{16}	D	uvr3:UVR3-2	1.76E-04	1.76E-04	1.76E-04	1.00E+00	1.00E-00	1.76E-04	6.97E-02	1.76E-04	1.88E-04	1.76E-04	9.87E-01	1.00E-00	1.00E+00	1.00E-00	9.94E-01		1.00E-00	1.00E-00	1.00E+00	1.00E+00
{17}	D	uvr3:UVR3-6	1.76E-04	1.76E-04	1.76E-04	1.00E-00	9.95E-01	1.76E-04	2.28E-01	1.76E-04	2.07E-04	1.76E-04	6.58E-01	1.00E-00	1.00E-00	9.60E-01	5.31E-01	1.00E-00		1.00E-00	1.00E-00	1.00E-00
{18}	D	uvr3:UVR3-7	1.76E-04	1.76E-04	1.76E-04	1.00E-00	1.00E-00	1.76E-04	2.20E-01	1.76E-04	2.71E-04	1.76E-04	8.65E-01	1.00E+00	1.00E-00	9.99E-01	9.06E-01	1.00E-00	1.00E-00		1.00E-00	1.00E-00
{19}	D	uvr3:UVR3-11	1.76E-04	1.76E-04	1.76E-04	1.00E+00	1.00E-00	1.76E-04	1.26E-02	1.76E-04	1.76E-04	1.76E-04	9.95E-01	1.00E-00	1.00E+00	1.00E-00	9.93E-01	1.00E+00	1.00E-00	1.00E-00		1.00E+00
{20}	D	uvr3:UVR3-12	1.76E-04	1.76E-04	1.76E-04	1.00E+00	1.00E-00	1.76E-04	1.03E-02	1.76E-04	1.76E-04	1.76E-04	9.97E-01	1.00E-00	1.00E+00	1.00E-00	9.95E-01	1.00E+00	1.00E-00	1.00E-00	1.00E+00	

Table S7. The ANOVA summary of all effects tested.

Effect	Sum of squares	Degrees of freedom	MS	F	p
intercept	41.30326	1	41.30326	5199.241	0.00
Plant line	2.84457	9	0.31606	39.786	0.00
Repair conditions	5.64435	1	5.64435	710.508	0.00
Plant line x Repair condition	2.14223	9	0.23803	29.963	0.00
Error	0.60375	76	0.00794		

Video S1. Time series of confocal images of the lower epidermis guard cells of the *uvr3*:AtUVR3GFP-6 line. The series duration is 1 min 38 s. The argon-ion 488 nm laser line was used for excitation. The fluorescence in the 494 - 597 nm range was recorded as the green channel. The series was recorded with the PlanApo 63x, NA 1.4 objective.

Supplementary references:

Sage D, Donati L, Soulez F, Fortun D, Schmit G, Seitz A, Guiet R, C. Vonesch, M. Unser. DeconvolutionLab2: An Open-Source Software for Deconvolution Microscopy Methods - Image Processing for Biologists, 115,. 2017
