# Supplementary material for: The photoreactivation of 6 − 4 photoproducts in chloroplast and nuclear DNA depends on the amount of the Arabidopsis UV repair defective 3 protein
Source: BMC Plant Biol. 2024 Jul 30;24:723. doi: 10.1186/s12870-024-05439-0 (PMC11287969; doi:10.1186/s12870-024-05439-0)
Supplement: Supplementary file 3 — Supplementary Material 3 [file 12870_2024_5439_MOESM3_ESM.pptx]

## Slide 1
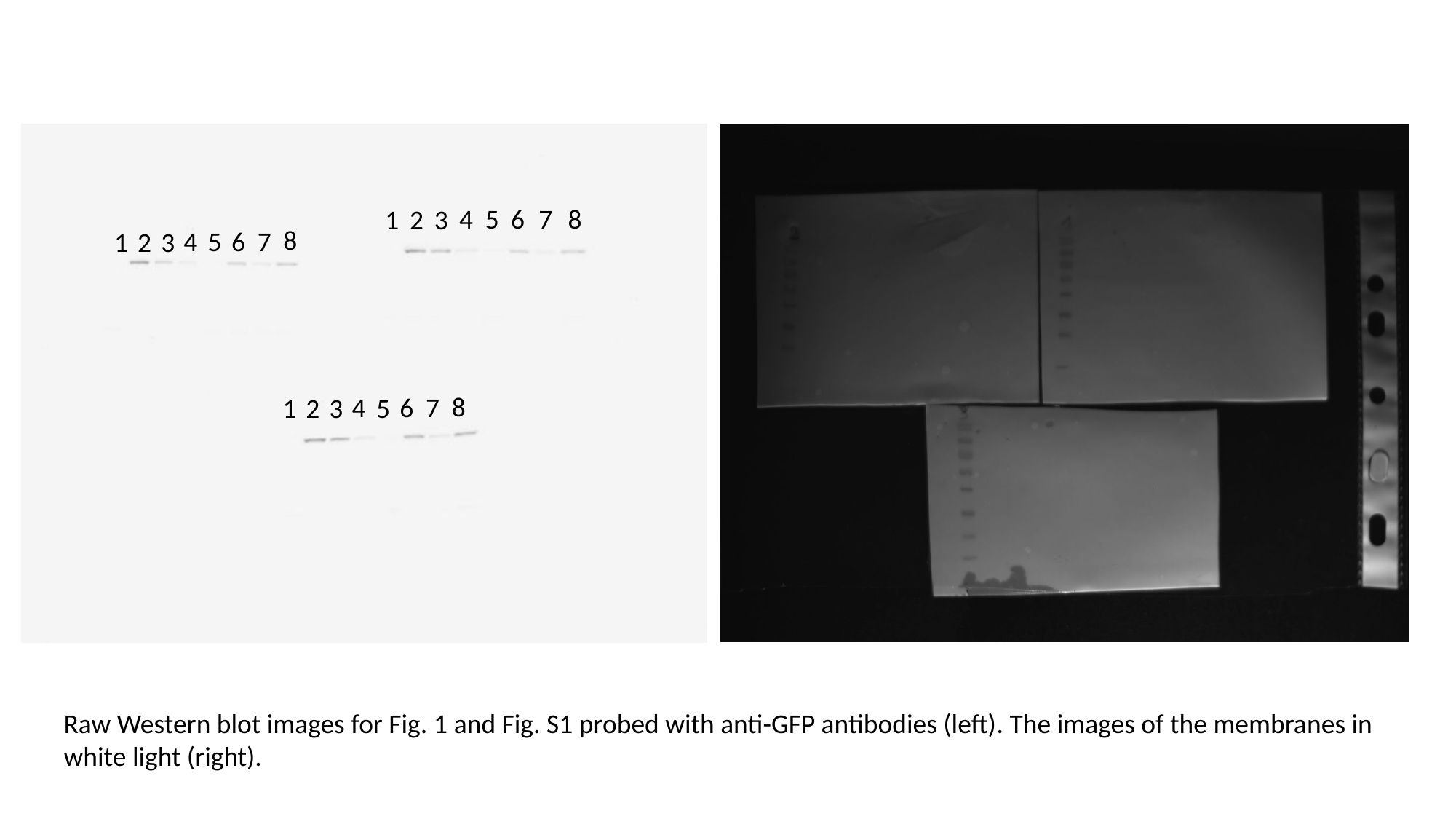

5
6
7
8
4
1
2
3
8
7
4
6
5
1
2
3
8
7
4
6
5
1
2
3
Raw Western blot images for Fig. 1 and Fig. S1 probed with anti-GFP antibodies (left). The images of the membranes in white light (right).

## Slide 2
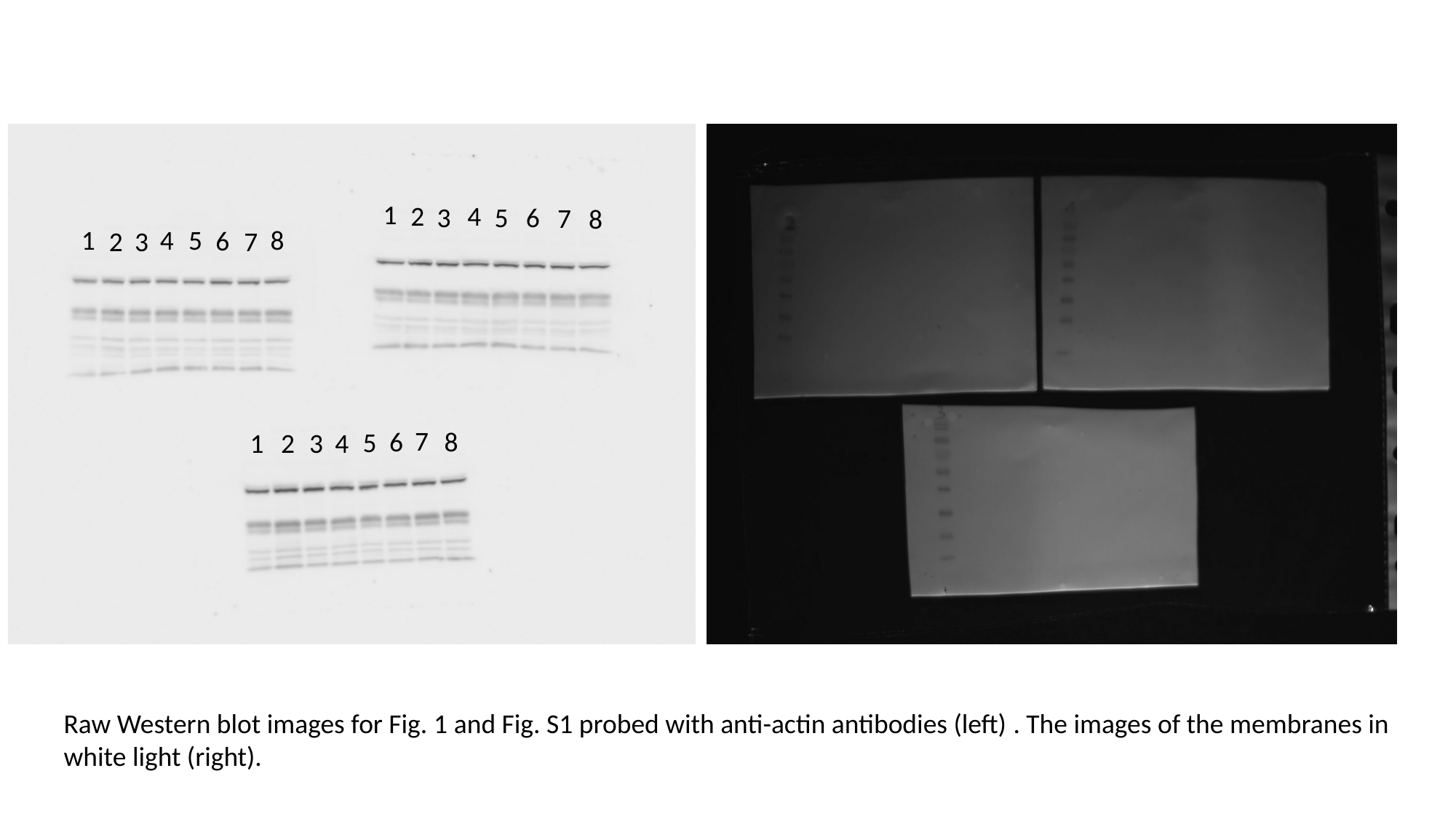

1
2
4
3
5
6
7
8
1
4
8
5
6
2
3
7
7
6
8
5
2
3
4
1
Raw Western blot images for Fig. 1 and Fig. S1 probed with anti-actin antibodies (left) . The images of the membranes in white light (right).
